# The conservation and evolutionary modularity of metabolism

**DOI:** 10.1186/gb-2009-10-6-r63

**Published:** 2009-06-12

**Authors:** José M Peregrín-Alvarez, Chris Sanford, John Parkinson

**Affiliations:** 1 Program in Molecular Structure and Function, Hospital for Sick Children, College Street, Toronto, ON, M5G 1L7, Canada; 2 Department of Molecular Biology and Biochemistry, University of Malaga, Avda. Cevantes, 29071 Malaga, Spain; 3 Department of Molecular Genetics, University of Toronto, King's College Circle, Toronto, ON, M5S 1A8, Canada; 4 Department of Biochemistry, University of Toronto, Toronto, Kings' College Circle, ON, M5S 1A8, Canada

## Abstract

**Background:**

Cellular metabolism is a fundamental biological system consisting of myriads of enzymatic reactions that together fulfill the basic requirements of life. The recent availability of vast amounts of sequence data from diverse sets of organisms provides an opportunity to systematically examine metabolism from a comparative perspective. Here we supplement existing genome and protein resources with partial genome datasets derived from 193 eukaryotes to present a comprehensive survey of the conservation of metabolism across 26 taxa representing the three domains of life.

**Results:**

In general, metabolic enzymes are highly conserved. However, organizing these enzymes within the context of functional pathways revealed a spectrum of conservation from those that are highly conserved (for example, carbohydrate, energy, amino acid and nucleotide metabolism enzymes) to those specific to individual taxa (for example, those involved in glycan metabolism and secondary metabolite pathways). Applying a novel co-conservation analysis, KEGG defined pathways did not generally display evolutionary coherence. Instead, such modularity appears restricted to smaller subsets of enzymes. Expanding analyses to a global metabolic network revealed a highly conserved, but nonetheless flexible, 'core' of enzymes largely involved in multiple reactions across different pathways. Enzymes and pathways associated with the periphery of this network were less well conserved and associated with taxon-specific innovations.

**Conclusions:**

These findings point to an emerging picture in which a core of enzyme activities involving amino acid, energy, carbohydrate and lipid metabolism have evolved to provide the basic functions required for life. However, the precise complement of enzymes associated within this core for each species is flexible.

## Abstract 2

A novel evolutionary analysis of metabolic networks across 26 taxa reveals a highly-conserved but flexible core of metabolic enzymes.

## Background

Cellular metabolism, represented by complements of enzymatic and transport reactions, is a fundamental biological system required for sustaining life. With a strong tradition in biochemistry, metabolism is probably the most widely characterized biological system studied to date. Several resources are now available that detail the metabolic complement of a wide range of organisms [1,2]. With the exception of a few model organisms, for the most part these are derived through automated methods capable of reconstructing metabolic networks through sequence similarity searches of known enzymes against their genomes [1-8]. Representation of metabolism is typically provided in the form of pathway maps that collate enzymes on the basis of their involvement in related biochemical conversions [1,2]. However, given that many pathways share common enzymes, metabolism is increasingly being viewed as an assemblage of functional modules, interconnected through common enzymes and substrates that together coordinate a cell's biochemical activities. This treatment of metabolism as a single integrated network allows the application of sophisticated graph theoretical analyses to uncover fundamental organizational principals within the network. For example, it has been suggested that metabolism displays the typical scale free behavior of small world networks [9-11] (that is, most of the enzymes within the network have only a few connections while a few enzymes, so called hubs, are very highly connected), although this has been questioned in a more recent study [12].

Recently, with the availability of increasing numbers of genomes, there has been considerable interest in examining biological networks from an evolutionary perspective [13-16]. Applied to metabolism, such studies are beginning to yield insights into the effects of selection pressures at both local (pathway) and global (network) scales. Recent studies suggest a 'core' set of metabolic reactions, conserved across many organisms, highlighting the fundamental role of metabolism that is subject to strong evolutionary constraint [17-19]. However, aside from the core, there is increasing evidence that pathway expansions have evolved through the recruitment and/or loss of functional modules of enzymes with related activities [17,18,20]. The ability of a functional module to evolve may play a key role in developing or limiting biological robustness [21]. For example, evolutionarily 'frozen' modules may be less able to withstand errors, such as mutational inactivation of gene products, within the same functional module. Focusing on metabolism, two recent studies have used Jaccard coefficients (JCs) to measure the similarity of phylogenetic profiles and identify modules of evolutionarily related enzymes [16,22]. Yamada and colleagues [16], for example, identified over 200 'phylogenetic network modules' consisting of enzymes proposed to behave in a similar way in the evolutionary process of the metabolic network. Additional studies have further suggested that enzyme modularity is affected by both environmental factors and evolutionary relationships [23,24]. For example, Kreimer and colleagues [23] noted a trend of modularity decrease from ancestors to descendants. Together these studies suggest the need to explore modularity from the perspective of known phylogenetic relationships.

Due to the paucity of available eukaryotic genomes (to date the full genome sequence of only approximately 60 eukaryotes are currently available [25]), previous comparative studies of metabolism have largely focused on prokaryotes. Recently, we have collated and processed expressed sequence tag datasets to generate so called 'partial genomes' for almost 200 eukaryotes, including members of underrepresented taxonomic classes such as plants, nematodes and various groups of protists [26]. These data represent a valuable sequence resource that provides a unique opportunity to perform more comprehensive studies of metabolic pathways within Eukaryotes, allowing comparisons to be placed within a detailed taxonomic context. The inclusion of these expressed sequence tag datasets allows us to build further on previous studies of metabolic conservation by providing greater confidence to determining the taxonomic coverage afforded by individual enzymes as well as pathways. We highlight enzymes and pathways associated with taxonomic innovations and examine the evolution of these metabolic processes in the context of a novel approach to examining enzyme co-conservation.

## Results

### Conservation of enzyme activities over the three domains of life

Collating 2.7 million sequences associated with 193 partial genomes [26,27], 167 fully sequenced genomes and the non-redundant protein database (***nr***), we performed a systematic scan to detect potential homologs of 29,893 proteins representing 1,474 distinct enzyme activities (Table 1; Additional data files 1 and 2). The accurate annotation of catalogs of enzymes from genome sequences remains a significant challenge [28]. Due to the scale of this analysis and consistent with other genome annotation schemes [2,18,29,30] we employed BLAST as an efficient means of inferring the conservation of the 1,474 enzyme activities. It is appreciated that the use of BLAST can lead to a high rate of false positive assignments. For example, the well annotated genomes of *Escherchia coli *strain O157:H7 and *Saccharomyces cerevisiae *have 567 and 426 enzymes, respectively, curated in the Kyoto Encyclopedia of Genes and Genomes (KEGG) database resource [2]. With a bit score cutoff of 50 (equivalent to an E-value of approximately e-5), BLAST identifies an additional 512 enzymes for *E. coli *and an additional 501 enzymes for *S. cerevisiae*, resulting in a false positive rate of approximately 50%. On the other hand, the rate of false negatives is less than 2%. Increasing the cutoff to 100 (equivalent to an E-value of approximately e-20) was found to lower the false positive rate to only approximately 42%. Aside from BLAST, profile-based methods have also been employed [6,31]. However while these methods significantly reduce the incidence of false positives, they also result in a high incidence of false negatives. Finally, it is worth noting that approaches based on orthology mappings, such as that employed by KEGG, can greatly improve on BLAST annotations. However, such approaches are limited to organisms with complete genome sequences and may be problematic where such sequences are highly divergent. Here the aim is to incorporate non-genome sequence data to provide a more global survey of enzyme conservation. The effect of using BLAST with a relatively flexible score cutoff will result in an over-prediction of enzyme conservation, but has the benefit of providing highly conservative estimates of which enzymes and pathways are likely absent from specific taxa. Consequently, care must be taken when interpreting the results of these analyses.

**Table 1:** Number and sources of genomes and sequences used in this study broken down into taxonomic categories

Domain	Taxonomic grouping	Partial genomes	Partial genome sequences	Complete genomes	Complete genome sequences	***nr ***sequences	Total sequences
Archaea	Crenarchaeota	-	-	4	11,120	12,339	23,459
Archaea	Euryarchaeota	-	-	14	30,396	38,863	69,259
Archaea	Archaea - Other	-	-	1	563	3,180	3,743
Archaea	Total	-	-	19	42,079	54,382	96,461
							
Bacteria	Actinobacteridae	-	-	14	49,608	68,041	117,649
Bacteria	Alphaproteobacteria	-	-	14	48,997	81,233	130,230
Bacteria	Betaproteobacteria	-	-	9	37,184	51,947	89,131
Bacteria	Gammaproteobacteria	-	-	27	94,933	188,458	283,391
Bacteria	Deltaproteobacteria	-	-	4	13,778	15,449	29,227
Bacteria	Epsilonproteobacteria	-	-	4	7,128	16,452	23,580
Bacteria	Cyanobacteria	-	-	6	20,983	32,380	53,363
Bacteria	Firmicutes	-	-	31	72,975	163,215	236,190
Bacteria	Spirochaetes	-	-	4	10,163	18,324	28,487
Bacteria	Bacteria - Other	-	-	14	36,760	61,550	98,310
Bacteria	Total	-	-	127	392,509	697,049	1,089,558
							
Eukarya	Protist - Alveolata	10	29,707	2	8,691	24,211	62,609
Eukarya	Protist - Euglenozoa/Haptophyceae/Stramenophiles	7	13,846	1*	11,397*	9,484	34,727
Eukarya	Protist - Other	-	-	-	-	12,862	12,862
Eukarya	Protists - Total	17	43,553	3	20,088	46,557	110,198
Eukarya	Fungi - Ascomycota	17	44,358	9	52,271	67,765	164,394
Eukarya	Fungi - Basidiomycota	7	14,785	1^†^	431^†^	10,264	25,049
Eukarya	Fungi - Glomeromycota/Zygomycota	3	3,398	-	-	734	4,132
Eukarya	Fungi - Other	-	-	1	1,996	2,558	4,554
Eukarya	Fungi - Total	27	62,541	10	52,271	78,763	193,575
Eukarya	Metazoa - Lophotrochozoa	4	14,631	-	-	12,416	27,047
Eukarya	Metazoa - Arthropods/Tardigrades	17	22,528	2	33,585	95,953	152,066
Eukarya	Metazoa - Deuterostomes	21	90,244	2	57,406	276,682	424,332
Eukarya	Metazoa - Nematoda	34	95,345	2	39,464	38,657	173,466
Eukarya	Metazoa - Other	-	-	-	-	3,424	3,424
Eukarya	Metazoa - Total	76	222,748	6	130,455	427,132	780,335
Eukarya	Plantae	76	221,896	2	30,533	190,711	443,140
Eukarya	Total	196	550,738	21	233,347	743,163	1,527,248
							
**Total**		**196**	**550,738**	**167**	**667,935**	**1,494,594**	**2,713,267**

All partial genome sequences were obtained from PartiGeneDB [26]. Complete genome sequences refer to protein coding sequences obtained from the COGENT database [56] with the exception of those marked with an asterix, which represents the genome of *Thalassiosira pseudonana*, obtained from the Joint Genome Institute [58], and those marked with a dagger, which represent the genome contigs of *Coprinopsis cinerea*, obtained from the Broad Institute [59].

**Figure 1:**
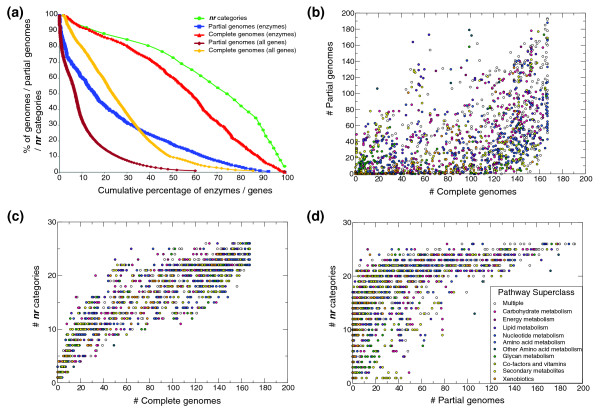
Representation of enzymes within three large-scale datasets. **(a) **Coverage of enzymes and genes provided by the three different datasets: the non-redundant protein database (***nr***); partial genomes; and complete genomes. Fifty percent of all enzymes are associated with approximately 15% of all partial genomes, approximately 60% of all complete genomes and approximately 75% of the ***nr ***categories used in this study. Compared to all the genes within the partial and complete genome datasets, the enzymes are more highly represented. **(b) **Relationships of enzyme coverage between the partial and complete genome datasets. Each point indicates a discrete enzyme (color indicates superclass membership - see inset key in (d)). Enzymes involved in secondary metabolism appear to be more highly represented in the partial genome datasets than the complete genome datasets. **(c,d) **As for (b) but showing the relationship of enzyme coverage between the ***nr ***dataset and the complete and partial genome datasets, respectively.

In the following we use the term 'enzyme' to refer to a collection of isoforms associated with a single enzyme activity (defined by unique Enzyme Commission (EC) numbers). To explore the distribution of enzymes across a broad set of informative taxonomic groupings, we defined 26 distinct taxa on the basis of number of species, sequence coverage and diversity (Table 1). These taxa are broadly consistent with previous studies of molecular evolution across the three domains of life [32]. Of the 1,474 enzymes studied here, half possessed significant sequence similarity in at least 60% of the completely sequenced genomes (complete genomes) and at least 17% of the partial genomes (Figure 1a; Additional data file 3). The lower observed incidence of detectable enzymes in the partial genomes reflects the incomplete nature of these datasets. In terms of taxonomic distribution, 190 enzymes (13%) were detected in 25 of our 26 defined taxa (Table 1); 933 (63%) enzymes were detected in all six major taxonomic groups (defined as: Archaea, Bacteria, Protists, Fungi, Metazoa and Plants); and 1,145 (78%) enzymes were detected in each of the three domains of life (Archaea, Bacteria and Eukarya) (Figure S1 in Additional data file 1). Overall, metabolic enzyme sequences were found to be more highly conserved than randomly selected sets of proteins from ***nr ***(Figure S2a in Additional data file 1), consistent with previous findings [18].

### Relationships between enzyme function and patterns of conservation

In addition to organizing enzymes into 118 distinct biochemical pathways, the KEGG database (version 33) [2] groups these pathways into ten functional subcategories (which we term 'superclasses'): carbohydrate metabolism; energy metabolism; lipid metabolism; nucleotide metabolism; amino acid metabolism; other amino acid metabolism; glycan metabolism; co-factors and vitamins; secondary metabolites; and xenobiotics. Enzyme conservation was therefore examined in the context of enzyme superclasses (Figure 1b-d; Additional data file 3).

Enzymes involved in multiple metabolic superclasses had significantly higher rates of detection in both complete and partial genomes (median number of complete/partial genomes = 126/57) compared with all other enzymes (median number of complete/partial genomes = 93/30; Mann-Whitney test, *P *< 10^-5 ^for both genome datasets). Conversely, those involved in glycan metabolism (median complete/partial genomes = 19/14; Mann-Whitney test, *P *< 10^-5 ^for both genome datasets) and xenobiotics (median complete/partial genomes = 72.5/7; Mann-Whitney test, *P *< 5 × 10^-5 ^for complete genomes, *P *< 10^-5 ^for partial genomes) were significantly less conserved in both genome datasets.

Between the datasets some notable differences in enzyme conservation were observed (Additional data file 3). Specific to the complete genome datasets, enzymes involved in nucleotide metabolism had significantly higher rates of detection (median number of genomes = 127; Mann-Whitney test, *P *< 10^-4^), while enzymes involved in secondary metabolites had significantly lower rates of detection (median number of genomes = 24; Mann-Whitney test, *P *< 10^-5^). Within the partial genome datasets, enzymes involved in lipid metabolism had significantly higher rates of detection (median number of partial genomes = 41; Mann-Whitney test, *P *< 0.01). Interestingly, enzymes associated with co-factors and vitamins had significantly higher rates of detection in the complete genomes (median number of complete genomes = 116; Mann-Whitney test, *P *< 0.02), but significantly lower rates of detection in partial genomes (median number of partial genomes = 18; Mann-Whitney test, *P *< 10^-5^). Differences between the two datasets are also apparent from Figure 1b (note, for example, the higher incidence of enzymes associated with secondary metabolites and lipid metabolism in the partial genome datasets compared with the complete genome datasets). Overall, there was good correlation between enzyme conservation as measured by ***nr ***categories and complete genomes (Figure 1c; Additional data file 3). Interestingly many enzymes were identified as being restricted to specific domains of life. For example, 204 enzymes (14%) had homologs in Bacteria and Eukarya but appear absent from Archaea while 34 enzymes associated with 30 KEGG-defined pathways appear specific to bacteria (Additional data file 4). A detailed discussion of these findings is presented in Additional data file 1.

Complicating factors in interpreting these results include both the incomplete nature of the partial genome datasets and their bias towards highly expressed proteins (see Additional data file 1 for further discussion). However, in a previous study of global sequence diversity, we noted that findings obtained from the use of partial genome datasets were consistent with those obtained from the use of fully sequenced eukaryotic datasets [32]. Hence, we expect the observed differences between the complete and partial genome datasets likely reflect their taxonomic bias (complete genomes are mainly derived from prokaryotes, while partial genomes are derived solely from eukaryotes). In the following sections we explore these relationships in more detail.

### Conservation and taxonomic distribution of metabolic pathways

**Figure 2:**
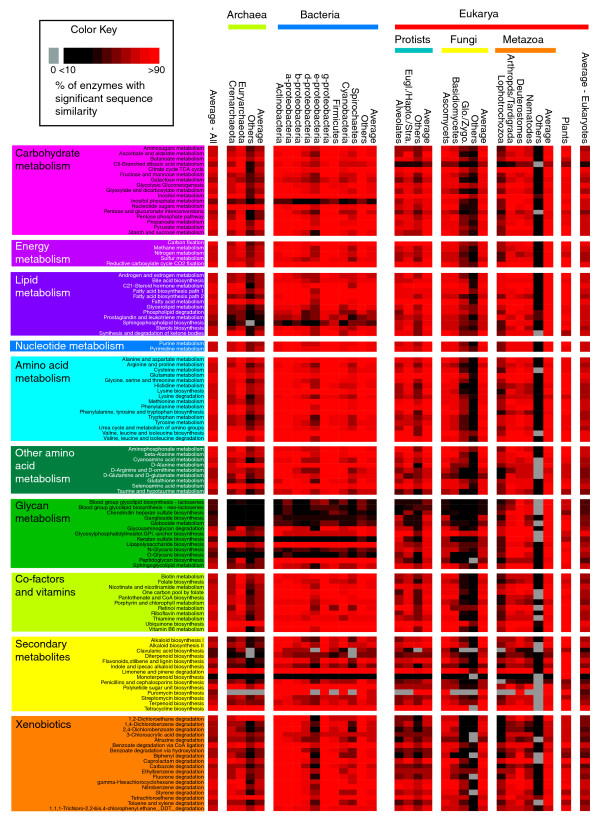
Heatmap showing the conservation of individual metabolic pathways. Each row indicates an individual metabolic pathway grouped by their superclass membership (defined by KEGG). Each column indicates specific taxonomic divisions (see Materials and methods; Table 1). Colored tiles indicate the level of conservation (percentage of enzymes detected) of each pathway within each taxonomic group (see inset color key top left). For example, many Glycan metabolic pathways are poorly conserved with the exception of several groups of metazoans.

In the previous section we examined the conservation of individual enzymes and found that, with a few notable exceptions, enzymes are broadly conserved across the three domains of life. Given that enzymes operate within the context of biochemical pathways, we next investigated the extent to which the pathways themselves are conserved. For each of the 118 pathways defined in KEGG, we identified the proportion of enzymes with homologs in each of the 26 taxonomic groups (Figure 2). For groups represented by fewer sequences (for example, lophotrochozoa and glomeromycetes/zygomycetes; Table 1; Figure S2c in Additional data file 1), metabolic pathways will consequently appear less well conserved. Nevertheless, from Figure 2 general trends in pathway conservation can be observed. Although pathways appear well conserved, 77 pathways having a mean percentage of conservation (MPC; the average percentage of enzymes detectable in each of the 26 defined taxonomic groups) of 70% or more (Additional data file 5), closer examination of conservation in the context of the three domains of life revealed that only 25 pathways had a MPC of 70% or more in each of Archaea, Bacteria and Eukarya. Five pathways were significantly more conserved than expected (*P *< 0.05 corrected for multiple testing - see Materials and methods): ubiquinone biosynthesis; fatty acid metabolism; glutamate metabolism; valine, leucine and isoleucine metabolism; and pyruvate metabolism. On the other hand, 11 pathways (all involved in glycan and secondary metabolism) had a MPC of less than 50%.

Across the 26 taxa, pathways display a spectrum of conservation, from those that are well conserved across all groups (for example, many pathways associated with carbohydrate, energy, nucleotide, amino acid, other amino acid and co-factor and vitamin metabolism) to those that are conserved in only a few specific taxonomic groups (for example, many glycan pathways are conserved only in metazoans) (Figure 2). These patterns are further emphasized through hierarchical clustering of the pathways based on their conservation profiles (Figure S3 in Additional data file 1). Such taxa-specific pathways represent unique innovations providing novel metabolic capabilities [33].

Pathways limited to specific domains include C5-branched dibasic acid metabolism, lipopolysaccharide biosynthesis and peptidoglycan biosynthesis, all poorly conserved in eukaryotes. The former pathway provides alternative sources of carbon and energy, while peptidoglycan and lipopolysaccharides are important components of the bacterial cell walls and envelope, respectively. On the other hand, inositol phosphate metabolism and sphingophospholipid biosynthesis were poorly conserved in prokaryotes, consistent with their roles as important components of secondary messenger systems and eukaryotic cell membranes [34-36]. Aside from these taxa-specific pathways, we noted that many pathways involved in secondary metabolism are more highly conserved in plants, ascomycetes and certain groups of bacteria such as the actinomycetes. Such pathways are associated with defense mechanisms (plants) [37], or as a means of surviving within a highly competitive environment (ascomycetes and bacteria) [38,39]. Finally, we note that many glycan biosynthetic pathways are restricted to a limited number of taxa, in particular the metazoa. This is consistent with the role of glycans in developmental and signaling processes associated with multicellularity [40,41].

### Identification of evolutionarily distinct modules of enzymes

From the preceding analysis it is not clear to what extent each pathway is conserved as a single entity. For example, pathways displaying moderate levels of conservation may arise from either a few organisms possessing the entire pathway, or many organisms possessing different, limited sets of enzymes. We therefore investigated the extent to which each pathway behaves as an evolutionarily distinct 'module' through the calculation of JCs (see Materials and methods). Due to the incomplete nature of the partial and ***nr ***datasets, modularity analyses were performed using the complete genome datasets only.

As for the conservation analyses, pathway modularity was calculated for a range of different taxonomic groups (Figure S4 in Additional data file 1; Additional data file 6). Similar to pathway conservation, we observe a well populated spectrum of pathway modularity with mean JCs (MJCs) for entire pathways ranging from 0.05 (type I polyketide biosynthesis) to 0.83 (inositol metabolism); where a MJC of 1.0 would indicate that the enzymes of an individual pathway are always present in the same genome. In addition, some pathways are more modular (higher MJC) in the context of specific taxa (for example, C21 steroid hormone metabolism has a MJC of 0.95 when considering only eukaryotic species, but a MJC of 0.60 when all species are considered. From Figure S4 in Additional data file 1, we see that many pathways involved in glycan metabolism (for example, glycosaminoglycan metabolism) are more modular in metazoans; certain pathways involving amino acids and lipids (for example, glutamate metabolism and fatty acid metabolism) are more modular in metazoans and plants; and several pathways involved in secondary metabolism (for example, clavulanic acid biosynthesis and diterpenoid biosynthesis) are more modular in plants and/or bacteria.

From these initial investigations we noted that pathways possessing enzymes that are highly conserved are likely to also have high raw JCs (Figure S5 and Results in Additional data file 1). We therefore assessed the significance of the observed modularity scores (as defined by MJC) through comparisons with 200 sets of randomly generated enzyme profiles. To account for biases in the phylogenetic relationships of the species sampled in this study (Figure S6 in Additional data file 1) [42], we applied a novel simulated annealing protocol to generate sets of adjusted enzyme profiles (see Materials and methods). These were used to obtain distributions of MJCs for each pathway allowing the calculation of Z-scores for the observed MJC (Additional data file 6). These provide a clearer view of which pathways may be defined as modular, accounting for biases in both enzyme conservation and evolutionary relationships of the organisms in which the component enzymes are found. It should be noted that the over-prediction of enzyme conservation discussed earlier has the potential to introduce noise into these analyses. Consequently, the modularity scores presented here should be treated as conservative estimates.

**Figure 3:**
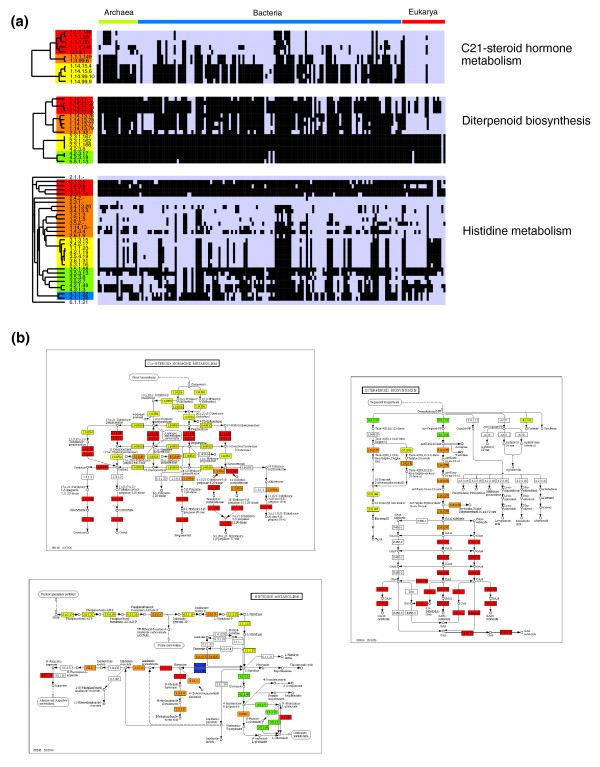
Representative examples of pathways containing evolutionary submodules of enzymes. **(a) **Clustergrams showing the phylogenetic profiles of individual enzymes in three metabolic pathways. For each clustergram, rows indicate individual enzymes and columns indicate individual genomes. A grey box indicates that the enzyme has been detected in that genome, black boxes indicate that it has not. Hierarchical clustering was performed using Cluster3.0 [70] using Spearman rank correlation coefficients and average linkage. Colored boxes indicate manually assigned groups of enzymes with similar phylogenetic profiles. **(b) **KEGG pathway representations of the three clustered pathways presented in (a). Enzymes are colored by the groups derived from (a). Within each pathway, groups of similar colored enzymes can be located to specific areas of each pathway, suggesting an evolutionarily cohesive module of function. For example, in diterpenoid biosynthesis, the red cluster of enzymes form a spatially distinct section of the pathway connected to the orange cluster of enzymes, while the green cluster of enzymes appears to form the beginnings of the pathway.

We found 49 pathways to be significantly modular (*P *< 0.01 corrected for multiple testing - see Materials and methods). These included many of the glycan metabolism pathways, several lipid pathways, monoterpenoid and diterpenoid biosynthesis, inositol phosphate metabolism, and phenylalanine and tyrosine metabolism. Many (26) of these were also associated with low MJCs (< 0.4), compared with only nine that had relatively high MJCs (> 0.6). In pathways with low MJCs, there was a significant enrichment in enzyme pairs with high JCs (> 0.6), suggesting that modularity arises through smaller sets of enzymes. To examine this in more detail, we selected three pathways with moderate to low MJCs but with a significant enrichment of high JCs (> 0.8). These were diterpenoid biosynthesis (MJC 0.27; Z-score = 17.4; 12% enzyme pairs with JC > 0.8); C21-steroid hormone metabolism (MJC 0.60; Z-score = 8.5; 26% enzyme pairs with JC > 0.8); and histidine metabolism (MJC 0.45; Z-score = 3.9; 12% enzyme pairs with JC > 0.8). For each pathway, genome profiles for each enzyme were clustered into distinct groups and mapped onto KEGG pathway maps (Figure 3). From the profiles of each group, it is clear how inclusion of all pairwise combinations of enzymes can result in a low to moderate pathway MJC (for example, note the large difference in the profiles of the enzymes in the groups colored yellow and orange in diterpenoid biosynthesis; Figure 3a). Figure 3b reveals that many of these groups representing evolutionarily distinct modules additionally represent limited but functionally cohesive units within their respective pathways.

### Metabolic network reconstruction and analysis

Metabolic pathways are typically defined through linked sequences of enzyme-catalyzed reactions with their boundaries determined through manual curation [43]. While such definitions can provide biological meaning, they tend to ignore the integrated nature of metabolism. Consequently, metabolism is increasingly being studied in the context of a single integrated network with nodes representing substrates and links between these nodes representing enzyme reactions (or vice versa) [4,10,16,17,22]. To examine evolutionary relationships outside pathway boundaries, we reconstructed a global metabolic network (with 1,329 nodes representing enzymes and 5,906 links representing common substrates) based on information of reaction pathways from KEGG. Consistent with previous studies [10], the network is scale-free (R = -0.92); that is, most enzymes have few links, while a few 'hub' enzymes possess many links. Note this network does not represent a true biological network in the sense of being specific to a single species, but rather represents the collection of metabolic pathways from across the three domains of life [16].

**Figure 4:**
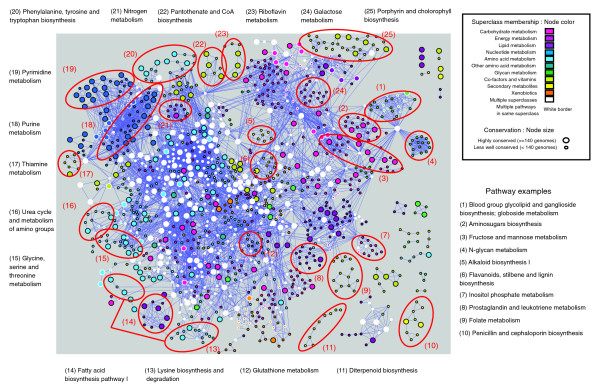
Conservation within the global metabolic network. An integrated view of metabolism in which individual enzymes (1,329 nodes) are connected through common metabolites (5,906 edges) (see Materials and methods). Colors of nodes represent which metabolic superclass (as defined by KEGG) each enzyme belongs to (see inset key). Node size indicates the number of genomes (of 167 complete genomes) in which the enzyme could be detected. A number of pathways with connected enzymes are indicated with red circles for illustrative purposes. While some nodes such as those involved in diterpenoid biosynthesis - pathway 11 - form a separate network, the vast bulk of metabolic pathways form connections with many others (for example, Nitrogen metabolism - pathway 21).

**Figure 5:**
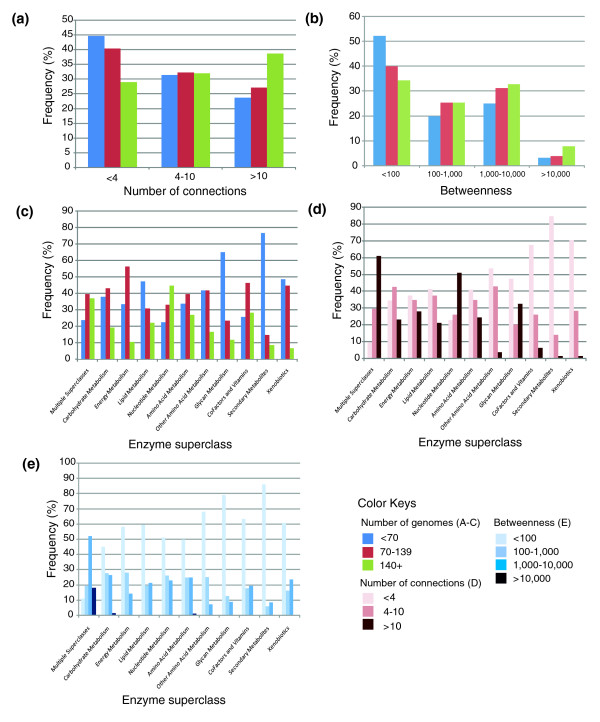
Metabolic network properties. The graphs indicate the relationships between enzyme superclass categories, conservation and connection within the metabolic network. **(a) **Number of connections as a function of enzyme conservation. **(b) **Centrality (as measured by betweenness) of enzymes as a function of conservation. **(c-e) **Enzyme superclass and its conservation, connection and centrality properties.

Visualization of the network (Figure 4) reveals the complex nature of this network. At the network periphery the distinct pathway structure of several pathways can be clearly discerned (for example, diterpenoid biosynthesis, penicillin and cephalosporin biosynthesis, folate metabolism, fatty acid biosynthesis pathway, N-glycan metabolism, and porphrin and chlorophyll biosynthesis). However, at the center of the network is a large 'core' of highly connected enzymes involved in multiple pathways, amino acid metabolism and carbohydrate metabolism. Many of these core enzymes are also highly conserved (Figure 5a). Since proteins with wider phylogenetic extent are expected to be of an older origin [18,44], this network core is likely associated with the ancestral form of the extant metabolic network.

We applied topological metrics in conjunction with their conservation profiles to analyze these relationships in more detail (Figure 5). Consistent with previous studies of biological networks [14,45], highly conserved enzymes (those identified in > 140 of 167 genomes) were more highly connected and more centrally located (higher values of betweenness) within the network (Figure 5a, b). They were also more likely to be involved in multiple KEGG-defined pathways (chi-squared score = 25.8; *P *< 0.001). Focusing on superclass designations, enzymes involved in multiple superclasses were also found to be highly connected (share many substrates) and more centrally located within the network than any other class of enzyme (Figure 5c-e). Enzymes involved in nucleotide metabolism were almost as highly connected but did not have similar high values of betweenness. This suggests that these pathways form highly integrated systems that operate at the periphery of the network. Along similar lines, enzymes involved in glycan metabolism, although poorly conserved, were also highly connected yet peripheral to the network (low betweenness values). Conversely, enzymes involved in xenobiotic metabolism, also poorly conserved, were not well connected and, intriguingly, had high betweenness values. Finally enzymes involved in secondary metabolism are poorly conserved, not well connected and peripheral to the network. The implications of these findings are presented in the Discussion.

We next examined co-conservation relationships (as defined by JC), between pairs of linked enzymes, within the context of the global network (Figure S7 in Additional data file 1). Using a cutoff Z-score of 2.0, 432 links involving 422 enzymes could be defined as significantly modular compared with the shuffled enzyme profiles (although as noted above, due to over prediction of enzyme conservation, this is likely to be a conservative estimate). Within this graph, many subnetworks consisting of co-conserved enzymes can be assembled into coherent pathways - mainly associated at the periphery of the network (for example, diterpenoid biosynthesis, N-glycan metabolism, folate biosynthesis and C21 steroid hormone metabolism). These pathways appear to provide specific functions requiring specialized enzymes that utilize unique substrates. For example, the main precursor in the diterpenoid biosynthetic pathway is geranyl geranyl pyrophosphate from which tree resin, plant hormones and anti-bacterial/fungal agents are synthesized.

Although co-conserved enzyme pairs associated with the core of the network are observed, it is interesting to note the relatively low ratio of links to enzymes (432:422) compared with the entire network (5,906:1,329). Hence, although many of these core enzymes tend to be highly connected, they tend only to be co-conserved with one or two other enzymes. The lack of complex networks of modularity within this core indicates that although the network core may represent the basic metabolic framework required for life, there is a degree of flexibility in the precise combination of enzymes.

## Discussion

Here we describe a systematic and comprehensive analysis of the conservation of the universal metabolic network (defined from the KEGG database) across a range of structural and taxonomic hierarchies. The inclusion of partial genomes provides a unique opportunity to extend our knowledge of the conservation of metabolism, particularly with respect to some of the more neglected Eukaryotic taxa (Figure S1 and Results in Additional data file 1). As such, this work builds on prior studies of metabolic conservation that were more restrictive in terms of sequence coverage and phylogenetic extent [18,33,46,47]. For example, Tanaka and colleagues [46] examined enzyme gain and loss events across six eukaryotes to reveal a gain in lipid metabolic processes in vertebrates. Similarly Freilich and colleagues [33] looked at the evolution of the human metabolic network from the perspective of vertebrate evolution. In the current paper, we provide a broader perspective and examine the conservation of enzymes and pathways across 26 taxa, selected on the basis of species coverage and evolutionary relationships [32].

Consistent with previous studies, we found enzymes involved in multiple superclasses were most highly conserved and those involved in glycan metabolism were least highly conserved [18]. Observed differences between the three datasets (complete genomes, partial genomes and ***nr***), revealed the bias towards prokaryotes in the complete genome datasets and highlight the need to consider integrative analyses such as these within a phylogenetic context. As noted earlier, the use of BLAST with a relatively flexible score cutoff will result in an over-prediction of enzymes. Consequently, the lack of homologs of enzymes involved in inositol phosphate and sphingophospholipid pathways in prokaryotes highlights the unique role of these pathways in the formation and function of lipid membranes associated with multi-compartment cells [36,48]. Similarly, many glycan biosynthetic pathways are largely restricted to the Metazoa. The expansion of glycan pathways in these organisms increases the repertoire of post-translational modifications, resulting in the production of proteins of greater complexity. This enables the addition of new functionalities and specificities that likely underlie the complex array of developmental and signaling processes that facilitate multi-cellular life [49].

**Figure 6:**
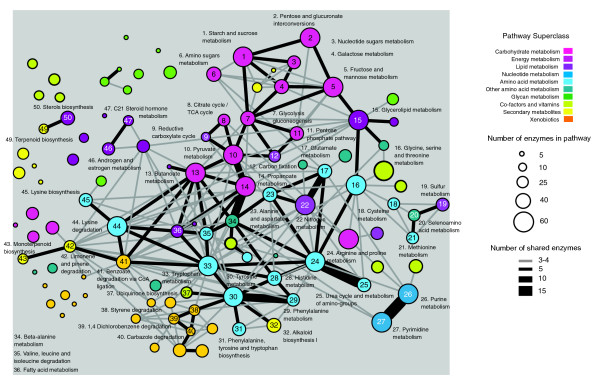
Crosstalk between metabolic pathways. The network diagram represents the number of enzymes shared between pathways. Each pathway is represented by a node. Connections (edges) between these nodes represent the number of enzymes common to each pathway. Nodes are colored according to their superclass category; node size indicates the number of enzymes in that pathway; and thickness of edges indicate the number of enzymes common to each pathway (see inset keys).

To circumvent biases that may occur in pathway definitions, recent studies of metabolic conservation are beginning to adopt a network approach in which metabolic components are linked as nodes in a graph [10,16,22,50]. The use of a global metabolic network map (Figure 4) allowed the identification of a highly interconnected core of conserved enzymes, many of which are involved in multiple pathways. Such enzymes support the notion that 'enzyme recruitment' plays a large role in metabolic evolution where novel pathways can emerge through the recruitment of enzymes (and hence their metabolites) from existing pathways [18,51]. This is more clearly seen in Figure 6, which shows the overlap of enzyme activities between different pathways. Pathways involving carbohydrate, amino acid and energy metabolism form a distinct core network with many shared enzyme activities. For example, pyruvate, butanoate and proponoate metabolism share large numbers of activities that are applied in slightly different contexts (for example, EC1.2.1.3, which represents a class of oxidoreductases with wide specificities). Tyrosine, tryptophan, phenylalanine and histidine pathways are also highly interlinked, presumably reflecting their common usage of aromatic substrates. Interestingly, xenobiotic pathways form their own interconnected cluster with a number of links (perhaps indicative of their origins) to the amino acid pathways: tyrosine and tryptophan metabolism and lysine degradation. On the other hand pathways involved in glycan metabolism and the generation of secondary metabolites are largely disconnected from the network, indicating their independent origins, perhaps from other processes not defined as part of metabolism. It should be noted that pathway overlap does not explicitly depict evolutionary relationships for the following reasons: firstly, enzyme activity relationships are independent of sequence relationships; and secondly, it is not clear to what extent pathway borders overlap. Nonetheless, these observations highlight the potential for enzymes to transcend pathway borders to facilitate new functions.

Previous studies examining the evolution of metabolism suggest the existence of functional modules of enzymes that share similar patterns of conservation and may act as evolutionary building blocks [16,22-24,52]. Two recent studies applied JCs to measure evolutionary modularity [16,22]. However, as we have shown here, the use of JC to infer modularity can be misleading if conservation is not taken into account: a high JC may merely reflect proteins that are highly conserved rather than mutual evolutionary trajectories. Furthermore, as noted by Kreimer and colleagues, evolutionary modularity can be influenced by both phylogenetic and environmental relationships [23]. Consequently, in our analyses, we developed a novel algorithm to generate simulated metabolic complements that reflect such relationships. Comparisons with these simulated datasets enable us to identify genuine instances of co-inheritance that do not simply reflect patterns of conservation and common evolutionary histories. For the most part, co-inheritance was restricted mainly to enzymes peripheral to the global metabolic network (for example, N-glycan metabolism and diterpenoid biosynthesis), and represent functions that support taxonomic innovations [53]. The relatively low number of links between co-inherited enzymes at the core of the network or in the nucleotide pathways indicates that despite such enzymes being very highly conserved, they do not always occur together in the same genomes. Given the highly connected nature of many of these proteins, we may further infer that hub proteins may only be co-conserved with a limited number of partners.

These findings point at an emerging picture in which a core of enzyme activities involving amino acid, energy, carbohydrate and lipid metabolism have evolved to provide the basic functions required for life. However, as indicated by the relatively low number of significantly modular links, the precise complement of enzymes associated within this core for each species is flexible. It is important to remember that the network view provided in Figure 4 represents a conglomeration of metabolic pathways from many different species. Subsequently, if we were to visualize any single species, we may expect to find a varying fraction of these core components missing. This is consistent with the idea that the large number of connections provided by these core enzymes might provide a wide variety of alternative routes for the production of key metabolites. The integration of poorly conserved enzymes and pathways involving xenobiotics within this core (high betweenness values) yet possessing low connectivities, suggests that they arise through the recruitment and divergence of existing enzymes from the network core. The periphery of the metabolic network consists of more recent enzyme innovations that, unlike the core enzymes, do not possess similar flexibility in the production of substrates. In the case of pathways involved in glycan metabolism, the large number of shared substrates, together with their relatively recent origin, suggests that they may have evolved from recent gene family expansion events resulting in a large number of enzymes sharing substrates. For example a number of reactions in the N-glycan biosynthetic pathway involve sequential additions of UDP-N-acetyl-D-glucosamine through a set of N-acetylglucosaminyltransferases. Pathways producing secondary metabolites are also of relatively recent origin but, unlike the glycan pathways, involve the use of novel substrates and consist of linear chains of reactions. Their modularity and lack of integration within the rest of the network may reflect the need to tightly control the production and conversion of pathway intermediates to ensure that potentially toxic products are not produced through exposure to unrelated enzymes.

These analyses provide indications on the adaptations to metabolic networks that have been acquired by different sets of organisms. Applied in the context of organisms of industrial importance, such findings could find application in metabolic engineering. For example, there is considerable interest in the production of the diterpenoid paclitaxel, an important anti-cancer therapeutic. Alternatively, applied to organisms associated with pathogenicity, these findings could be exploited for identifying pathways and enzymes that could be usefully targeted for therapeutic intervention (see Additional data file 1 for a simple example). Finally, the approaches outlined here have been limited to the study of metabolism, but may also be applied to other systems such as regulatory pathways (Additional data files 1 and 7).

## Conclusions

Here we have combined multiple sources of sequence data to perform a systematic and comprehensive analysis of the conservation and modularity of metabolism across the three domains of life. Intriguingly, while we identified a highly conserved set of enzymes at the core of the metabolic network, there appears to be enormous flexibility in their use across different organisms. It should be appreciated that in using BLAST to infer homology, this study does not claim to reconstruct the metabolic network of any single organism, but instead focuses on levels of sequence conservation associated with specific enzymes and pathways. As such a more detailed comparison of the metabolic networks encoded by individual species would help to further understand and interpret the biological meaning of the differences we have found. Given the scope of the analysis, we have been able to report only a small fraction of our findings. To facilitate further, in-depth explorations of the conservation and modularity of the metabolic network, we make the Cytoscape [54] files generated in this analysis freely available at [55].

## Materials and methods

### Sequence input data

Three types of sequence data were used in this study (see Table 1 and Additional data file 2 for details about the number of sequences, and taxonomic information on the genome datasets analyzed in this study). The first type comprised protein coding sequences of 167 complete genomes (19 Archaea, 127 Bacteria and 21 Eukarya) were derived from the COGENT database [56,57]. While 200 datasets were originally obtained, to avoid species redundancy in bacteria, if there were different strains from the same species, only one strain (the one with the largest number of open reading frames) was considered. In addition, we also obtained the protein coding sequences of *Thalassiosira pseudanana *[58] and the genome contigs of *Coprinopsis cinerea *[59]. The second type comprised the consensus DNA sequences associated with 193 eukaryotic partial genomes obtained from our in-house database, PartiGeneDB [26,60]. The third type comprised proteins from the non-redundant (***nr***) protein database SWALL (Swissprot and TrEmbl) [61].

Reference enzyme datasets were obtained from the KEGG database [2,62]. We obtained 29,893 sequences associated with 1,474 distinct EC numbers from 260 species (sequences with more than one EC number per sequence were excluded to reduce the number of potential false positive assignments when performing BLAST searches). In certain cases, we identified enzymes with EC numbers that were not assigned a sequence within the KEGG dataset. For example, according to KEGG, ten enzymes participate in the retinol metabolism pathway. However, we were only able to extract two of the sequences assigned to this pathway. Therefore, in a complementary approach, enzymes associated with EC numbers with no apparent sequence in the KEGG database were used as queries to search the ***nr ***database via the sequence retrieval system (SRS) [63]. This step has previously been successfully applied to identify missing genes in predicted metabolic pathway databases such as EcoCyc and MetaCyc [3] and was also found to extend the coverage of our approach by adding an additional 1,181 new protein sequences to the reference dataset.

### Taxonomic divisions

Species were classified into 26 taxonomic divisions on the basis of the NCBI taxonomy resource [64] (Table 1; and Additional data file 2). Each domain of life was split into the following major taxonomic divisions: Archaea (Crenarchaeaota, Euryarchaeota and Archaea_Others); Bacteria (Actinobacteridae, Alphaproteobacteria, Betaproteobacteria, Gammaproteobacteria, Deltaproteobacteria, Epsilonproteobacteria, Cyanobacteria, Firmicutes, Spirochaetes and Bacteria_others); and Eukarya (Protists, further subdivided into Alveolata, Euglenozoa/Haptophyceae/Stramenophiles and Protist_Others; Fungi, further subdivided into Ascomycota, Basidiomycota, Glomeromycota/Zygomycotina and Fungi_Others; Metazoa, further subdivided into Annelida/Mollusca/Platyhelminthes, Arthropoda/Tardigrada, Chordata/Echinodermata, Nematoda and Metazoa_Others; and Plantae). Note that both the Euglenozoa/Haptophyceae/Stramenophiles group and the eukaryotic groups specified as 'Others' represent paraphylyetic groups and, therefore, artificially grouped together for convenience.

### Conservation of metabolic enzymes and pathways

The process for obtaining the metabolic complement for each species and taxonomic divisions was as follows. First, we applied BLAST [65] to identify sequences with significant sequence similarity (defined using a raw bit score threshold of 50 and E-value of 10^-5^) to 29,893 metabolic enzymes from the KEGG dataset and the additional 1,181 enzymes derived through the SRS procedure (see above) from the previously defined taxonomic partitions. The above threshold was considered to be a useful compromise between the rate of discovery of false negatives and false positives and is similar to the threshold adopted in previous studies [18,46]. This process results in a taxonomic profile for each of the 1,474 EC numbers analyzed in this study, displaying the presence or absence of a sequence sharing significant sequence similarity with an enzyme with the appropriate EC number within each of the 167 complete genome datasets, 196 partial genome datasets and the ***nr ***protein dataset (divided into the 26 aforementioned taxonomic divisions). Note that these profiles are different to the phylogenetic profiles traditionally applied to individual proteins, as they represent a consensus 'EC profile' that may have been derived through many different isoenzymes.

The MPC of each pathway (MPC_ij_) was calculated by:

(1)

where e_ij _is the number of isoenzymes in the jth pathway with homologs in the ith taxonomic group, E_j _the total number of isoenzymes with sequence in the jth pathway according to KEGG, and N is the number of taxonomic groups analyzed. Since the pathway content we are analyzing is based on the number of enzymes (reactions) involved in metabolic pathways, multiple isoenzymes catalyzing the same reaction were counted only once, and multifunctional enzymes were counted as many times as they catalyze different reactions. To assign levels of significance associated with the conservation of each pathway, we compared the observed MPC for each pathway with the MPCs derived from 10,000 pathways of equivalent-sized pathways generated through random sampling of enzymes. *P*-value thresholds associated with the derived Z-scores were then adjusted using the Bonferroni correction for multiple testing. For 116 pathways examined in the conservation analyses, a conventional *P*-value of 0.05 is corrected to an α-score of 4 × 10^-4^, resulting in a critical Z-score of 3.33. This approach is similar to that described by Lopez-Bigas and colleagues [66].

To assess whether the observed phylogenetic distribution patterns of metabolic enzymes were different from any other proteins, 25 sets of proteins of equal size to the enzyme dataset were randomly taken from proteins from the ***nr ***database with no EC numbers in their annotations (that is, potentially representing unknown metabolic enzymes) as control sets, and were subjected to an identical analysis. For the statistical analysis we used a two-tailed *t*-test at 99% confidence level.

### Evolutionary modularity

The evolutionary modularity of metabolic pathways was quantified using only sequence data from complete genomes. Briefly, we calculated the relative distance of the phylogenetic profiles for pairs of enzymes [67]. For each pair of enzymes we calculated the JC, an established measure of similarity between two phylogenetic profiles [16,68]; a JC of 1 indicates 100% overlap, and a JC of 0 indicates no similarity. Finally, we assessed pathway modularity using both approaches by calculating the MJC of all pairs of enzymes considered for the pathway as follows:



where JC_ij _is the JC measure of each pair of enzymes involved in the jth pathway, and N is the number of distinct enzyme pairs known to participate in the jth pathway. By definition, a pathway is perfectly modular (100%) if all enzymes within the pathway have exactly the same phylogenetic profile (either present or absent in a given species; that is, the modularity should be 1).

To ascribe a level of significance to the calculated JCs, we created 200 sets of randomly assigned enzyme profiles. These were created to reflect the observed distribution of enzyme profiles using an approach based on simulated annealing (Additional data file 6). Three properties were measured from the original profile: number of genomes each enzyme is found in, number of enzymes each genome contains and the similarity of each genome with each other. Prior to annealing, each genome was initialized with the correct number of enzymes, but shuffled randomly. During each annealing step an enzyme was selected from one species, and swapped with a different species. The change in scoring function was evaluated and the swap accepted or rejected with the probability:



Where ΔE is an energy term associated with the change in scoring function (see the following equation) and T is a temperature term associated with the simulated annealing protocol:



where δs is the sum of the Hamming distances between the target similarity matrix (a matrix of Hamming distances between the enzyme profiles of every pair of genomes - effectively providing a measure of the relatedness of enzyme content between organisms) and the current matrix, Sw (= 1) is a weight associated with this first term, δe is the Hamming distance between the target number of genomes each enzyme is found in and the current matrix, and Ew (= 15) is a weight associated with this second term. The temperature term, T, was initialized to 1,000 and decreased in a non-uniform adaptive fashion to 0.00001. Temperature was decreased to 0.9 of its previous value only after 10^6 ^consecutive swap attempts failed to improve the overall score. The procedure was re-run for 200 different initially assigned profiles. This resulted in sets of enzyme profiles in which, although the specific enzyme membership for each genome was shuffled, the similarity between the enzyme content from closely related species was maintained.

### Metabolic network reconstruction and analysis

Several methods and databases are available to reconstruct an organism's metabolic network from genome information, such as KEGG [2] and MetaCyc [1]. In this study, we reconstructed and represented the complete metabolic network as an undirected enzyme (or reaction) interaction graph. In this graph, enzymes (EC numbers) are represented as nodes and substrates connecting two enzymes are represented as edges in the network. Common metabolites were ignored, consistent with previous studies [4]. Pathway relationships were inferred from KEGG. Certain enzymes can catalyze several different reactions and are involved in different metabolic pathways. These enzymes are represented by single nodes in the network. Thus, these nodes are linked to all other nodes that are connected to the different reactions or pathways undertaken by these enzymes. Network properties (node degree and betweenness values) were obtained using the tYNA network analysis platform [69].

## Abbreviations

EC: Enzyme Commission; JC: Jaccard coefficient; KEGG: Kyoto Encyclopedia of Genes and Genomes; MJC: mean Jaccard coefficient; MPC: mean percentage of conservation; ***nr***: non-redundant protein database; SRS: sequence retrieval system.

## Authors' contributions

JMPA helped conceive and design the study, collated and analyzed sequence datasets and drafted the manuscript. CS developed the statistical framework for accounting for phylogenetic bias in analyzing JCs. JP conceived and designed the study, performed additional statistical analyses and wrote the paper.

## Additional data files

The following additional data files are available with the online version of this paper: supplemental material in terms of results, discussion and methods and supplementary figures S1-S7 (Additional data file 1); a table listing the complete and partial genomes used in this study (Additional data file 2); a table listing the conservation of individual enzymes (Additional data file 3); a table listing enzymes restricted to either bacteria or eukaryotes (Additional data file 4); a table showing the conservation of 118 metabolic pathways analyzed in this study (Additional data file 5); a table listing the modularity of 166 metabolic pathways analyzed in this study (Additional data file 6); a table listing the conservation of 25 regulatory pathways defined by KEGG (Additional data file 7).

## Supplementary Material

Additional data file 1 Supplemental results, discussion, methods and Figures S1-S7. Click here for file

Additional data file 2 The complete and partial genomes used in this study. Click here for file

Additional data file 3 Conservation of individual enzymes. Click here for file

Additional data file 4 Enzymes restricted to either bacteria or eukaryotes. Click here for file

Additional data file 5 Conservation of 118 metabolic pathways analyzed in this study. Click here for file

Additional data file 6 Modularity of 166 metabolic pathways analyzed in this study. Click here for file

Additional data file 7 Conservation of 25 regulatory pathways defined by KEGG. Click here for file
