# Supplementary material for: The conservation and evolutionary modularity of metabolism
Source: Genome Biol. 2009 Jun 12;10(6):R63. doi: 10.1186/gb-2009-10-6-r63 (PMC2718497; doi:10.1186/gb-2009-10-6-r63)
Supplement: Additional data file 1 — Supplemental results, discussion, methods and Figures S1-S7. [file gb-2009-10-6-r63-S1.pdf]

Supplementary information

Supplementary results

Sequence coverage provided by partial genome (EST based) datasets

Expressed sequence tags (ESTs) are single pass reads from randomly selected cDNA libraries. To create so called partial genomes, we have applied a clustering algorithm which groups ESTs on the basis of sequence similarity [26]. These are used to construct gene objects that may not necessarily be full length. For example, we note that the average length of a consensus sequence for the 32 nematodes used in this study was 481 nucleotides. This compares to an average length of 441 amino acid residues for proteins derived from *Caenorhabdidtis elegans*. Across the 193 genomes, the average number of gene objects was 2,842 +/- 2,291, with a maximum of 11,409 (*Allium cepa*) and a minimum of 209 (*Zeldia punctata* see Additional data file 2). Depending on the organism, 2,842 genes can represent from ~15% of a genome (e.g. Nematodes) to ~50% of a genome (e.g. Protists). Finally it is noted that unless normalized [71], EST-based datasets are typically biased towards transcripts of high abundance. Together these factors will impact the inference of metabolic enzymes from any single EST dataset as demonstrated by the lower coverage of partial genomes for each enzyme in Additional data file 3. Nevertheless, the grouping of individual species-datasets together on the basis of taxonomic relationships provides an informative view of enzyme and pathway conservation across the eukarya. For example from figure S2C, we note an asymptotic relationship between the number of enzymes detected and the number of sequences analysed, suggesting that sampling ~60-100,000 sequences is likely sufficient to inform on much of the metabolic capability of a particular taxon. Furthermore, although more sequences appear to have been sampled for deuterostomes and plants, they were found to possess fewer enzymes than several groups of bacteria, with upto one fifth of the sampled sequences. Interestingly, the Alveolates which include many parasites were found to possess fewer enzymes than several other groups which had fewer sequences such as the other group of protists – euglenozoa, haptophycea and stramenopiles. This might reflect previous findings of reduced metabolic capability associated with some of these organisms [72].

Integration of the three sequence datasets increases enzyme coverage

To examine the impact of combining all three datasets (***nr*** database, partial and complete genomes) to assess enzyme conservation, we assessed their independent coverage together with the coverage provided by their integration (Fig. S1A). Data integration produced a significant increase in the number of conserved enzymes compared to the independent use of any of the three data sets (NB partial genomes can only assess coverage for the eukaryotic species). For example only 5% of enzymes could be defined as ***nearly ubiquitous*** (present in 25 of the 26 defined phyla) when only complete genome data was used. However, when all three datasets are considered 13% of enzymes could be defined as ***nearly ubiquitous***. These results demonstrate the complementary nature of the three datasets and the increased taxonomic coverage provided through their integration. Indeed, the inclusion of the ~200 partial genome datasets provides a breadth and depth of taxonomic coverage within the Eukarya that is currently not possible if only species with complete genomes are considered [26, 32]. For example, use of the partial genomes broadened the definition taxonomic divisions (for example, Glomeromycota/Zygomycota - see

Table 1), and enhance the possibility of identifying enzyme isoforms in otherwise sparsely populated phyla. Likewise, the integration of complete genome information complements the Eukaryotic partial genomes through the addition of Archaeal and Bacterial datasets. Finally the inclusion of data from the ***nr*** database increases the number of species across all three domains of life.

Domain-specific enzymes

Many enzymes were identified as being restricted to specific domains of life (Fig. S2B and Additional data file 4). For example, 204 enzymes (14%) were found to be present in Bacteria and Eukarya but absent from Archaea. 34 enzymes associated with 30 KEGG defined pathways were identified to be specific to bacteria. Of these ten were associated with carbohydrate metabolism, while another seven were associated with xenobiotics or secondary metabolic pathways. These likely represent domain-specific metabolic capabilities. For example: clavaminate synthase (EC1.14.11.21) catalyses three of the reactions in the pathway required for the biosynthesis of the anti-bacterial agent – clavulanic acid (a beta lactamase inhibitor); 2-dehydro-3-deoxygalactonokinase (EC2.1.7.58) provides an alternative mechanism for catabolizing D-galactose and has been implicated in the breakdown of exogenous galactonate in those organisms in which both mechanisms are present [73]; and oligogalacturonide lyase (EC 4.2.2.6) and 4-deoxy-L-threo-5-hexulose uronate isomerase (EC 5.3.1.17) have been implicated in the degradation of pectin by plant pathogens [74].

Of the 64 enzymes specific to Eukaryotes, nine were associated with carbohydrate metabolism, 23 with glycan metabolism and 14 with secondary metabolites. Unlike the bacterial-specific enzymes, many of these eukaryotic-specific enzymes appear in the same pathway. For example six are associated with the inositol phosphate pathway, eight are associated with N-Glycan biosynthesis and four are associated with diterpenoid biosynthesis. Interestingly o-pyrocatechuate decarboxylase (EC 4.1.1.46) was found only in ascomycetes and is involved in the conversion of anthranilate and 2-aminobenzoate to catechol via 2,3 dihydroxybenzoate. In an alternative pathway, anthranilic hydroxylase found in bacteria and other eukaryotes, converts anthranilate and 2-aminobenzoate directly. These results suggest that while the evolution of eukaryotes did not require a large recruitment of novel reactions, several new pathways (e.g. inositol phosphate metabolism, glycan metabolism and secondary metabolism) have arisen in this lineage.

It is perhaps surprising that no Archaea-specific enzymes were detected (Fig. S2A), this may be a consequence either of the smaller number of sequences of the Archaeal datasets and/or the lack of studies devoted to Archaeal metabolism.

Not all highly conserved pathways possess high modularity and vice versa

Comparisons of pathway conservation (as measured by MPC – see methods) and modularity (as measured by MJC – see methods), revealed a high correlation (R=0.67) as noted in the main text (Fig. S5). However, some interesting exceptions are worth mentioning (see Additional data file 6). The monoterpenoid biosynthesis pathway is not highly conserved (MPC = 22%), with constituent enzymes being largely restricted to plant species, but is relatively modular (MJC = 0.60). These findings highlight the fact that a majority of enzymes in this pathway are always required to be present in order to produce the highly specialized product that is largely restricted to plants. On the other hand, the phospholipid degradation and retinol metabolism pathways are both highly conserved (MPC = 70% and 77% respectively) but with

low values of modularity (MJC = 0.27 and 0.20 respectively). This low modularity suggests a degree of flexibility associated with these pathways. Either different organisms require only a fraction of the functionality provided by the entire pathways, or the pathways contain alternative modes of operation performed by limited subsets of enzymes. Interestingly, many pathways involved in glycan metabolism are poorly conserved but also highly modular. The observed relationships between conservation and modularity suggest that these parameters may, in principle, be exploited for purposes of predicting functionality of novel unannotated pathways or functional modules.

Conservation and evolutionary modularity of regulatory pathways

Although not reported in the main text, we performed a preliminary analysis of the conservation and modularity of a limited set of regulatory pathways obtained from KEGG (see Additional data file 7). Our initial results indicate a much lower level of conservation and modularity within these pathways. Of the 1,571 regulatory proteins extracted from KEGG, only 14 (~1%) could be defined as ***nearly ubiquitous*** (present in 25 of the 26 defined taxa). Being more flexible in the criteria of conservation, 278 (~18%) were present in all 6 major taxonomic groups, and 297 (~19%) were present in all three domains of life. Interestingly, these values are even lower than those obtained from 25 sets of randomly selected proteins derived from the ***nr*** database (MPC=26% - Fig S1C). The participation of regulatory proteins in pathways was similarly poorly conserved (see Additional data file 7). Only 4% (1/25) of the regulatory pathways with more than three enzymes had a MPC of 70% or more in each of Archaea, Bacteria and Eukarya (compared to 25/117 – 21% of metabolic pathways). However it should be pointed out that most (~70%) of the regulatory proteins were Eukarya specific including many signaling pathways associated with multicellular organisms. These findings highlight previous reports that the evolution of complexity and diversity is most likely a consequence of the diversification of regulatory mechanisms [75]. Evolutionary modularity of regulatory pathways was also analysed. Of the 26 regulatory pathways with more than three enzymes participating in the pathway, none were observed to have a MJC in excess of 0.7 (see Additional data file 7). This suggests that regulatory pathways are even less modular than metabolic pathways reflecting the high flexibility of regulatory processes.

Reconstruction of taxa-specific metabolic networks and prediction of potential drug targets.

There has been much recent interest in the reconstruction of metabolic networks for individual species for purposes of identifying potential drug targets [76-78]. Typically such reconstructions are performed through genome wide survey scans to identify the constituent enzymes. However errors in genome annotation or species-specific sequence diversification can lead to individual enzymes being missed [79]. Instead such analyses can benefit from adopting a taxon-wide approach in which a metabolic network is reconstructed for an entire phylum, minimizing the possibility of missing important players within the network. Subsequent experimental assays can then be applied to determine if the enzyme activity is indeed present in the target organism. To demonstrate how such an approach may be used, we reconstructed and analysed two taxon-specific metabolic networks. The first represents the Firmicutes, a bacterial taxon that includes many pathogens such as *Clostridium, Listeria, Mycoplasma, Staphylococcus,* and *Streptococcus*. The second represents the Alveolata, a group of related single celled parasites that include *Plasmodium* the causative agent of malaria. The Firmicute network comprises 1,104

enzymes involved in 5,056 functional interactions, while the Alveolate network comprises 873 enzymes involved in 3,435 functional interactions. Comparison of these networks indicate topological differences in their structure described by simple parameters like the number of nodes and interactions, which likely reflect their different evolutionary history, and probably their degree of metabolic dependency to the host. Other differences in the structure of the metabolic networks in the three domains of life has been previously shown [4, 9].

While more sophisticated approaches such as flux balance analysis have proved useful for purposes of identifying suitable therapeutic targets in metabolic networks [80-82], even simple metrics such as connectivity and degree of conservation of metabolic can help predict potential antimicrobial drug targets. For example, the enzymes protein-N(pi)-phosphohistidine-sugar phosphotransferase (EC 2.7.1.69), phosphate acetyltransferase (EC 2.3.1.8) and acetate kinase (EC 2.7.2.1) are all involved in carbohydrate metabolism and may represent potential antimicrobial drug targets due to: a) their level of conservation across most of Firmicutes and across many other organisms (more than 100); b) they are absent in human; and c) they appear to be highly connected in the reconstructed Firmicutes network, with 50, 21 and 13 connections, respectively. In the case of Alveolates, the enzymes phospholipase C (EC 3.1.4.3) and phosphoenolpyruvate carboxylase (EC 4.1.1.31) appear to be good potential antiparasite drug targets due to their degree of conservation across this taxa, absence in the human genome and their high connectivity, 15 and 13 connections, respectively. Furthermore, these five enzymes act as linkers in the network facilitating cross-talking between pathways. This suggests that they might be essential for the organism's biology and as such they may represent ideal antimicrobial drug targets.

Supplementary discussion

Assessment of sequence conservation is enhanced through considering taxonomic groups rather than individual species

During these analyses we have chosen to consider conservation and modularity in the context of taxonomic groups as opposed to individual species. As noted by Charlebois and Doolittle [79], analyses of conservation that rely on the analysis of genes across individual species can be prone to errors that arise from, for example, mis-annotation of genomes or species-specific sequence diversification. The grouping of species on the basis of shared taxonomic relationships and their use in assessing conservation helps minimize these effects which, as noted above can be useful for exploiting metabolic networks for the identification of useful drug targets. Furthermore, the inclusion of additional genomic datasets will increase, rather than decrease, our estimates of enzyme conservation across different taxonomic partitions.

The use of taxonomic groups also benefits attempts to identify the set of universal enzymes that reflects the basic metabolic complement required for life which might otherwise be reduced through the consideration of organisms with highly reduced genomes (e.g. parasites or symbionts). Such sets of sequences are indicative of the most ancient aspects of cellular biology, possibly present in the universal ancestor [83] and may be usefully exploited for deep phylogeny studies [84].

Metabolic pathways in the Archaea are less modular

Overall, we found that metabolic pathways in Archaea are less evolutionarily modular than in Eukarya and Bacteria. This is in agreement with findings suggesting that metabolic networks in non-Archaeal species are more clustered and modular than those in Archaeal species [9]. This low modularity reflects the both the exponential random-like and scale free behavior previously observed with the Archaeal metabolic networks which was suggested to be related to their adaptability to hostile environments [9]. However, these differences could be also due to the fact that metabolic pathways in Archaeal species has been much less studied [85].

Enzymes involved in multiple pathways may represent an ancestral form of the extant metabolic network

In the main text we have shown that enzymes involved in multiple pathways are both highly conserved and highly connected. These enzymes may be considered as important links that can mediate metabolic flux between pathways and responsible for making the network more interconnected. Elimination of these link enzymes would therefore be expected to greatly impact global metabolic flux supporting their need to be highly conserved. It has been suggested that ancestral enzymes may have been promiscuous to help adapting organisms to different environments [51, 86-88]. Duplication of these enzymes and their subsequent divergence may then have yielded enzymes with higher specificity resulting in an interconnected network of pathways. The finding that nearly a quarter of all metabolic enzymes are involved in multiple pathways further supports this “enzyme recruitment” scenario as a major force driving metabolic evolution [51] (see also main text).

Supplementary methods

Conservation of metabolic enzymes and pathways

KEGG is a knowledge based pathway database. As such some EC numbers may not have any sequence associated to them since some reactions (sequences) might have not been found in certain organisms. Thus, as a complementary approach, EC numbers without sequence assignments and those that had no hits to the above taxonomic divisions were used as queries to search the ***nr*** database using the sequence retrieval system (SRS - EBI, Cambridge – UK; http://srs.ebi.ac.uk). This step allows the extraction of sequences matching these unique EC numbers, and has previously been successfully applied to identify missing genes in predicted metabolic pathway databases such as EcoCyc and MetaCyc [1, 89]. This step extended the coverage of our approach by including in this analysis new sequences from other species (isozymes) associated with these KEGG EC numbers (1,181 new protein sequences in total). These sequences were used again as queries to search against different taxonomic partitions.

Taxa-specific network reconstructions

Using the complete metabolic network (incorporating current knowledge about enzymes participating in metabolic pathways according to KEGG) as a template, we identified the enzymes coded in an organism's genome using BLAST (see methods) and use this to reconstruct taxa-specific metabolic networks. In this analysis we focus in the metabolic capabilities of different taxa rather than individual species as previously noted.

Supplemental Figure Legends

Figure S1. The phylogenetic extent of metabolic enzymes

(A) For each enzyme in KEGG, BLAST was used to detect sequences with significant sequence similarity in four datasets: partial and complete genomes; a ***nr*** protein database; and an integrated set consisting of sequences from all three datasets. For each set we identify the frequency of enzymes associated with three different levels of conservation: those which are ***nearly ubiquitous*** (enzymes possessing significant sequence similarity to sequences from 25 of our 26 defined taxa - 13 out of 13 defined taxa for the partial genome dataset); those which are found in organisms representing all ***six major taxonomic groups*** (Archaea, Bacteria, Protists, Fungi, Metazoa and Plants – Note for the partial genome datasets the requirement was dropped to the four eukaryotic groups); and those which are found in all ***three domains of life*** (Archaea, Bacteria and Eukarya - just Eukarya for the partial genome dataset).

Figure S2. The phylogenetic extent of metabolic enzymes

(A) Conservation of metabolic enzymes compared to 25 control groups of 1,474 sequences randomly selected from ***nr***. Ss = species-specific proteins; A=Archaea; B=Bacteria, E=Eukarya; A_B_E=Archaea+Bacteria+Eukarya. All taxonomic partitions were significant compared with 25 sets of equal numbers of randomly selected proteins from ***nr*** ($p < 0.01$ two-tailed t-test). (B) Venn diagram showing the number of enzymes detected in each of the three domains of life. (C) Representation of enzymes within defined taxonomic groups. This graph indicates the number of enzymes (of 1,474 used in this study) detected in each of the 26 defined taxonomic groups as a function of the number of sequences associated with each of these groups.

Figure S3. Clustergram of relative metabolic pathway conservation

Conservation profiles consisting of the average percentage of enzymes found within 19 major taxonomic groups were constructed for each pathway. The conservation profiles were normalized and centered for each taxonomic group and clustered on their Pearson correlation coefficients using single linkage clustering. The clustergram thus indicates the relative conservation of each pathway within individual taxa. While similar to Figure 2, clustering allows the identification of groups of pathways (highlighted with surrounding coloured boxes) with similar conservation patterns. Such pathways may be indicative of taxon-specific innovations. For example a number of pathways (outlined by a green box) associated with secondary metabolites (diterpenoid biosynthesis; indole and ipecac alkaloid biosynthesis and monoterpenoid biosynthesis) are relatively poorly conserved across all taxa except plants.

Figure S4. Heatmap showing the evolutionary modularity of individual metabolic pathways

As for figure 2, rows and columns represent individual metabolic pathways (grouped by superclass membership) and taxonomic groups respectively. Coloured tiles indicate the level of

evolutionary modularity (average pathway Jaccard coefficient (JC) – see Methods) of each pathway within each taxonomic group (see inset colour key top left). For example enzymes involved in D-alanine metabolism are highly modular (high average JC) in the majority of bacterial groups but do not appear modular in the Metazoa. Only taxonomic groups with more than 2 complete genomes were considered (see Table 1).

Figure S5. Correlation between the evolutionary modularity of a metabolic pathway and its conservation

Each point indicates an individual metabolic pathway coloured according to its superclass category. Conservation score indicates the mean percentage of conservation (MPC - see Methods). Modularity score indicates the mean Jaccard coefficient (MJC - see Methods). Although there is a high correlation between pathway conservation and modularity, several pathways (mainly involved in Glycan biosynthesis) clearly do not share this trend.

Figure S6. Overlap of metabolic complements

The heatmap indicates the Hamming distance of the metabolic complements between pairs of organisms. A low Hamming distance indicates that the two organisms share very similar complements of enzymes, conversely a high Hamming distance indicates that the two organisms have very different complements of proteins. Note that certain groups of organisms have highly correlated complements of enzymes (e.g. the Mycoplasma's and the Fungi). Such correlations have been shown to interfere with phylogenetic profiling methods [42]. The order of species is indicated in Supplemental methods.

Figure S7. Evolutionary modularity within the metabolic network

As for figure 4, a metabolic network was constructed in which nodes represent enzymes and edges represent shared metabolites (excluding current metabolites – see Methods). Nodes are coloured by their superclass membership (see inset key). Here only enzymes with links deemed to be significantly evolutionarily modular are displayed (432 links, 422 enzymes). These are defined as links between two enzymes which have significantly overlapping phylogenetic profiles (Z-score > 2.0) compared with a control set of randomly generated profiles (normalized for close evolutionary dependencies). Several groups of enzymes representing distinct pathways are highlighted with coloured circles.

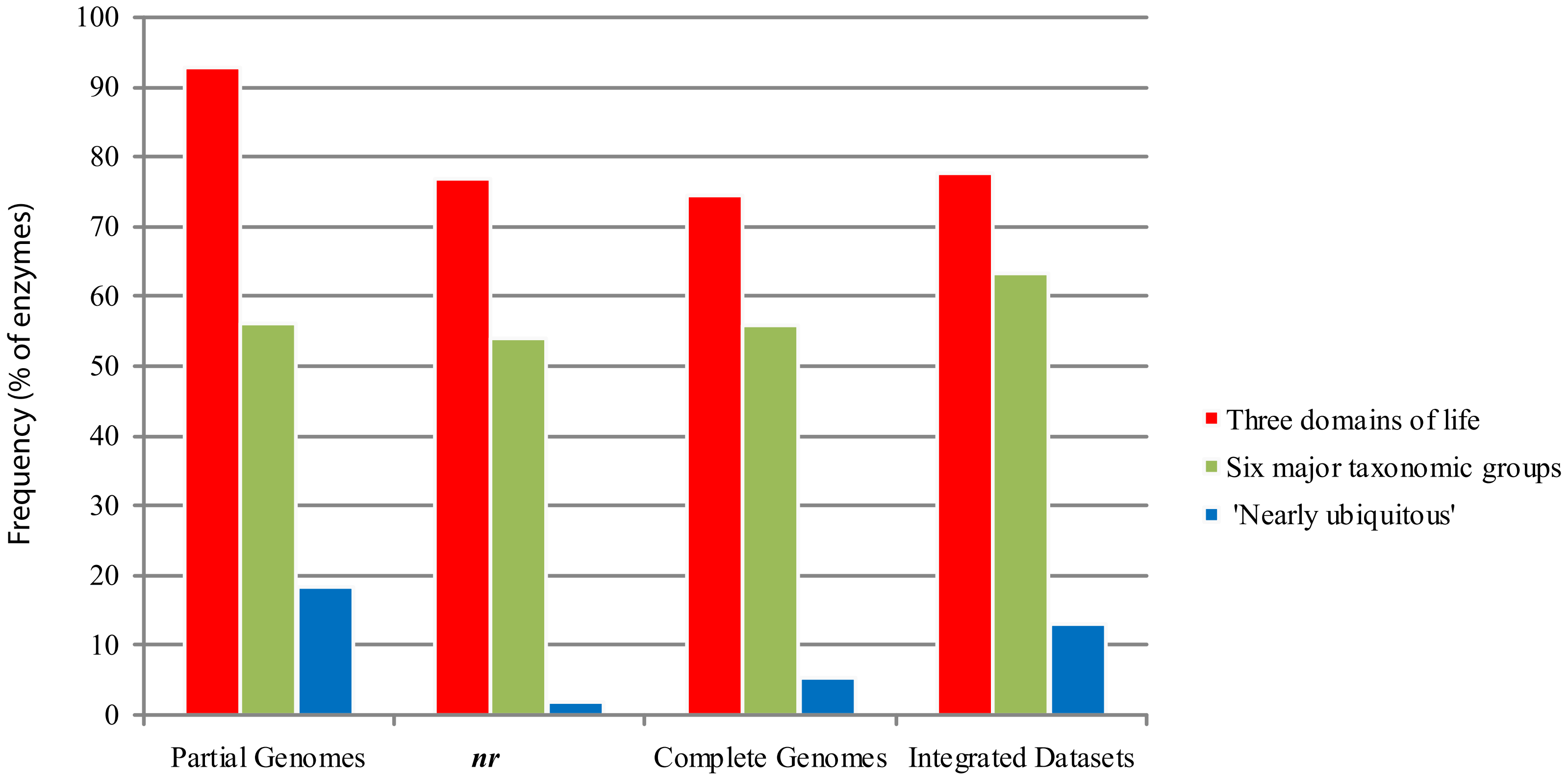
100
90
80
70
60
50
40
30
20
10
0
Frequency (% of enzymes)
Partial Genomes
nr
Complete Genomes
Integrated Datasets
Three domains of life
Six major taxonomic groups
'Nearly ubiquitous'

Fig. S1

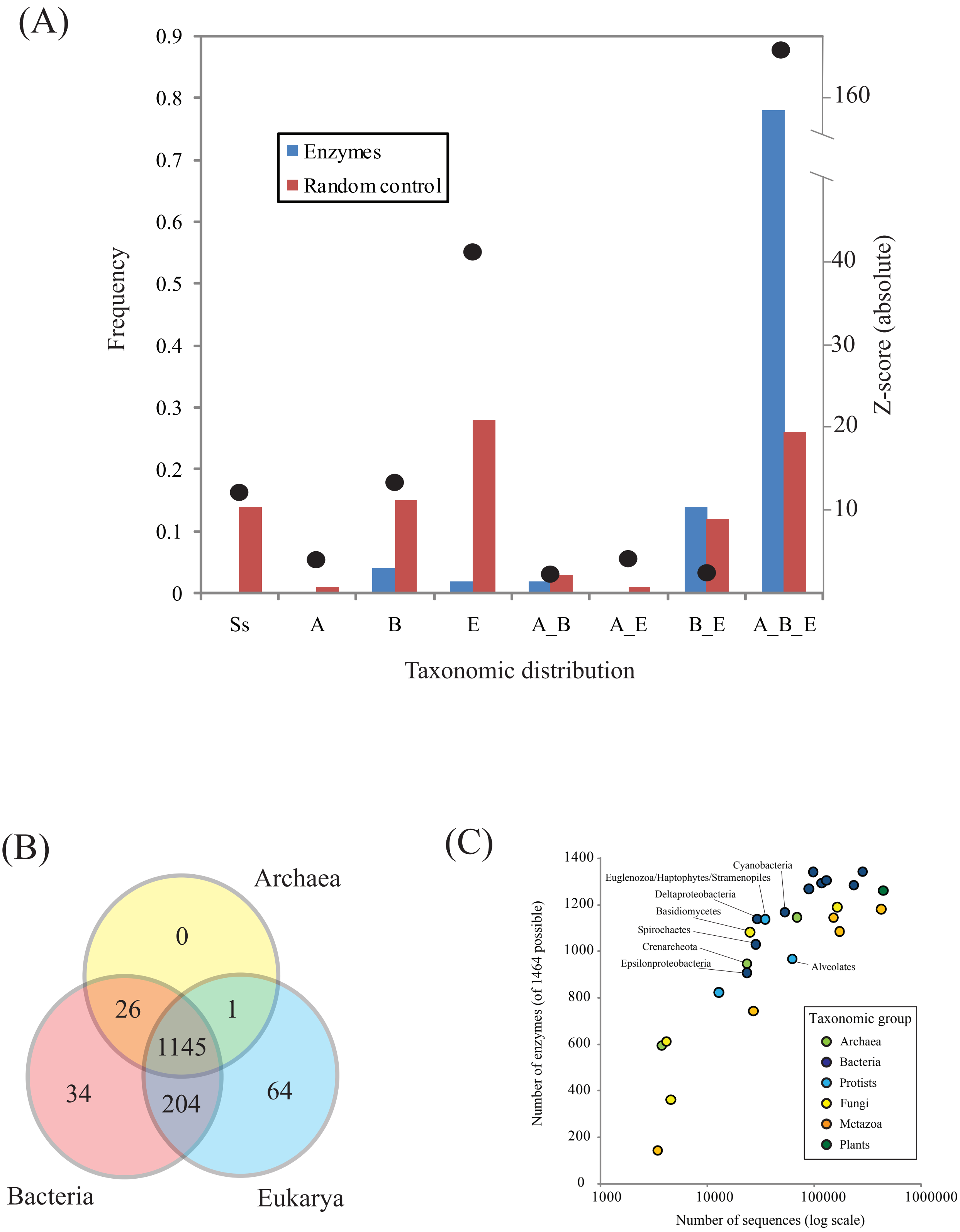

(A)
Enzymes
Random control
Frequency
Z-score (absolute)
Taxonomic distribution
Ss
A
B
E
A_B
A_E
B_E
A_B_E
(B)
Archaea
Bacteria
Eukarya
0
26
1
1145
34
204
64
(C)
Cyanobacteria
Euglenozoa/Haptophytes/Stramenopiles
Deltaproteobacteria
Basidiomycetes
Spirochaetes
Crenarcheota
Epsilonproteobacteria
Alveolates
Number of enzymes (of 1464 possible)
Number of sequences (log scale)
Taxonomic group
Archaea
Bacteria
Protists
Fungi
Metazoa
Plants

Fig. S2

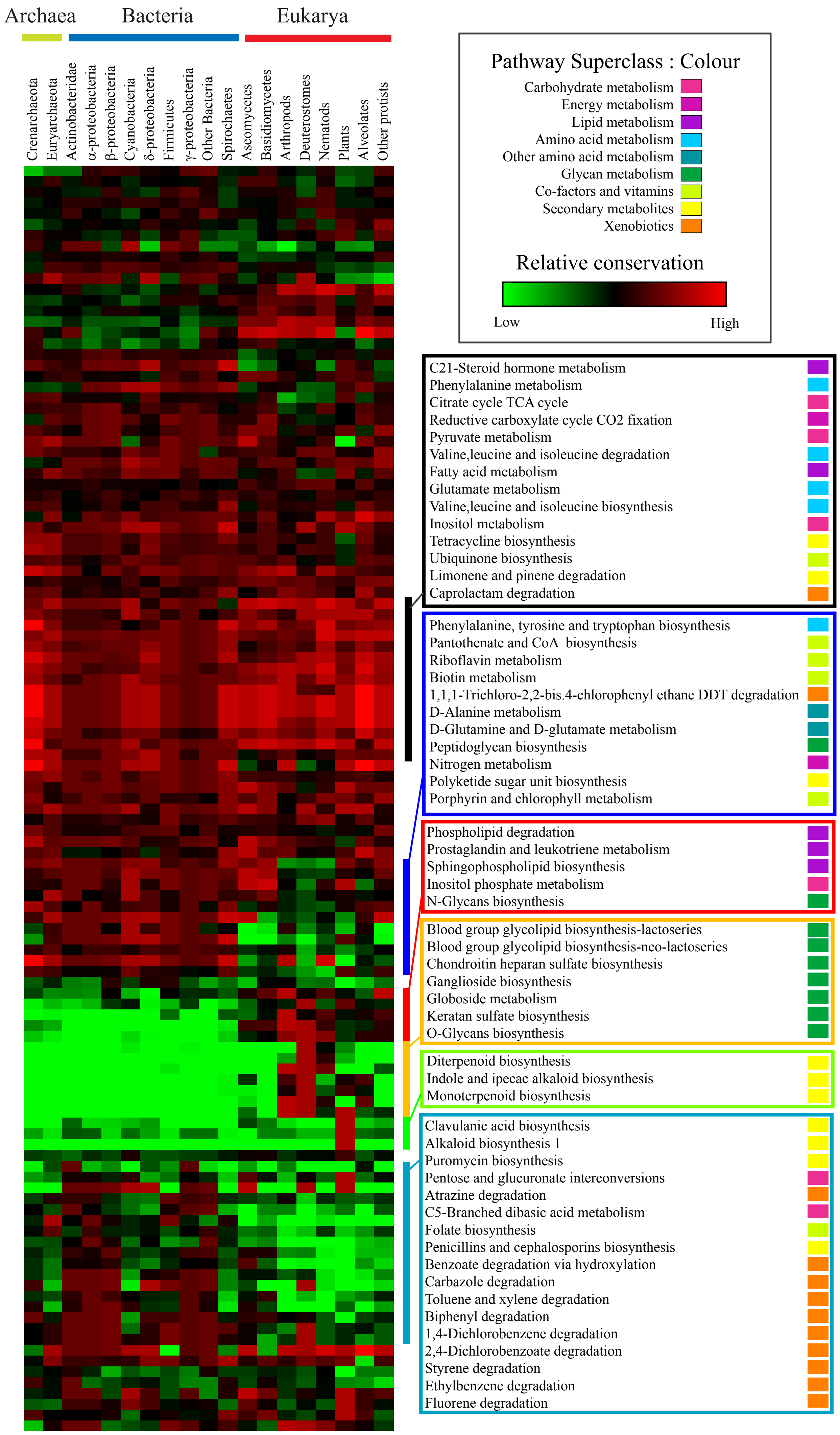
Archaea
Bacteria
Eukarya
Crenarchaeota
Euryarchaeota
Actinobacteridae
α-proteobacteria
β-proteobacteria
Cyanobacteria
δ-proteobacteria
Firmicutes
γ-proteobacteria
Other Bacteria
Spirochaetes
Ascomycetes
Basidiomycetes
Arthropods
Deuterostomes
Nematods
Plants
Alveolates
Other protists
Pathway Superclass : Colour
Carbohydrate metabolism
Energy metabolism
Lipid metabolism
Amino acid metabolism
Other amino acid metabolism
Glycan metabolism
Co-factors and vitamins
Secondary metabolites
Xenobiotics
Relative conservation
Low
High
C21-Steroid hormone metabolism
Phenylalanine metabolism
Citrate cycle TCA cycle
Reductive carboxylate cycle CO2 fixation
Pyruvate metabolism
Valine,leucine and isoleucine degradation
Fatty acid metabolism
Glutamate metabolism
Valine,leucine and isoleucine biosynthesis
Inositol metabolism
Tetracycline biosynthesis
Ubiquinone biosynthesis
Limonene and pinene degradation
Caprolactam degradation
Phenylalanine, tyrosine and tryptophan biosynthesis
Pantothenate and CoA biosynthesis
Riboflavin metabolism
Biotin metabolism
1,1,1-Trichloro-2,2-bis.4-chlorophenyl ethane DDT degradation
D-Alanine metabolism
D-Glutamine and D-glutamate metabolism
Peptidoglycan biosynthesis
Nitrogen metabolism
Polyketide sugar unit biosynthesis
Porphyrin and chlorophyll metabolism
Phospholipid degradation
Prostaglandin and leukotriene metabolism
Sphingophospholipid biosynthesis
Inositol phosphate metabolism
N-Glycans biosynthesis
Blood group glycolipid biosynthesis-lactoseries
Blood group glycolipid biosynthesis-neo-lactoseries
Chondroitin heparan sulfate biosynthesis
Ganglioside biosynthesis
Globoside metabolism
Keratan sulfate biosynthesis
O-Glycans biosynthesis
Diterpenoid biosynthesis
Indole and ipecac alkaloid biosynthesis
Monoterpenoid biosynthesis
Clavulanic acid biosynthesis
Alkaloid biosynthesis 1
Puromycin biosynthesis
Pentose and glucuronate interconversions
Atrazine degradation
C5-Branched dibasic acid metabolism
Folate biosynthesis
Penicillins and cephalosporins biosynthesis
Benzoate degradation via hydroxylation
Carbazole degradation
Toluene and xylene degradation
Biphenyl degradation
1,4-Dichlorobenzene degradation
2,4-Dichlorobenzoate degradation
Styrene degradation
Ethylbenzene degradation
Fluorene degradation

Fig. S3

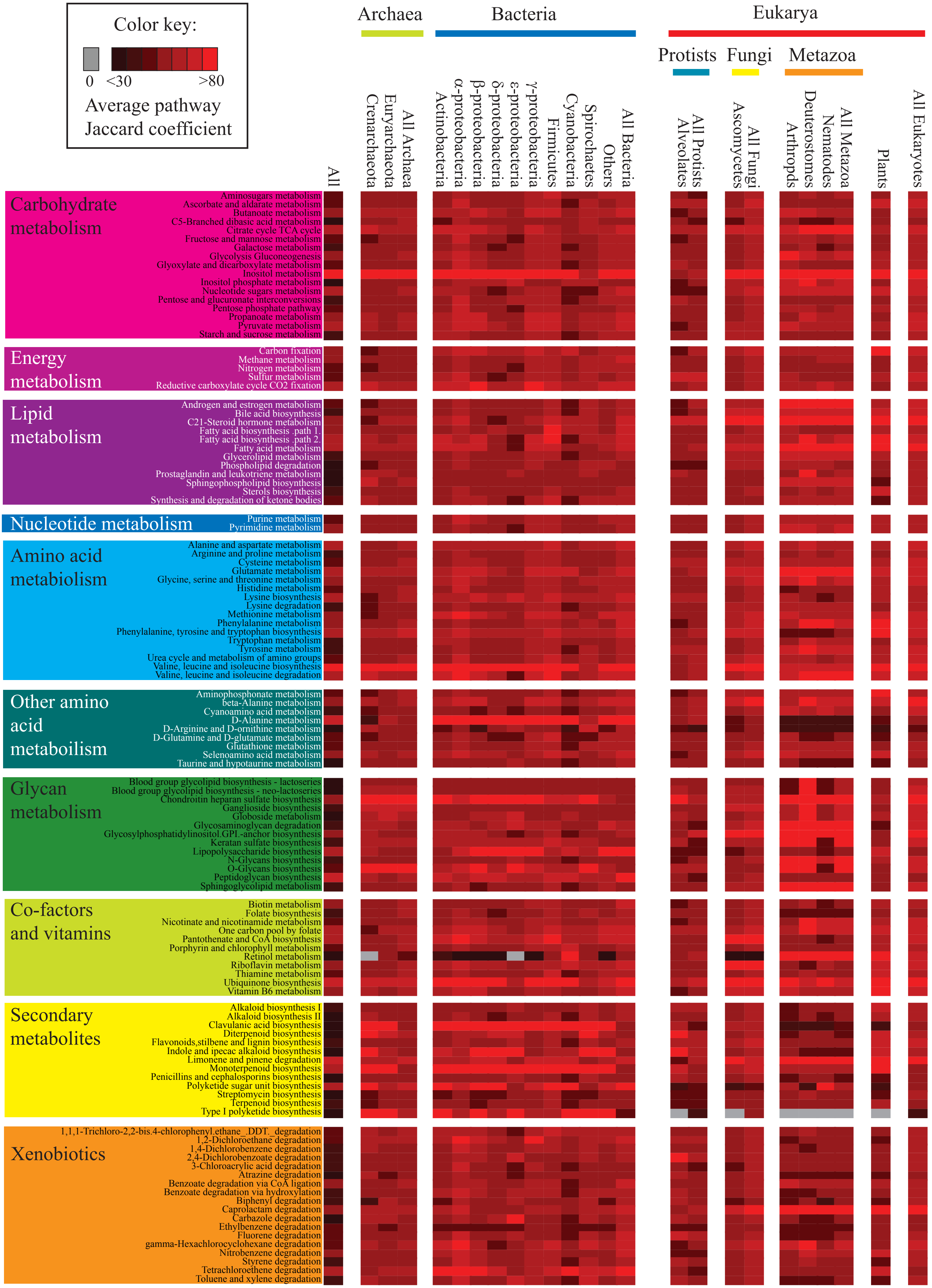

Color key:
0
<30
>80
Average pathway Jaccard coefficient
Archaea
Bacteria
Eukarya
Protists
Fungi
Metazoa
All
Crenarchaeota
Euryarchaeota
All Archaea
Actinobacteria
α-proteobacteria
β-proteobacteria
δ-proteobacteria
ε-proteobacteria
γ-proteobacteria
Firmicutes
Cyanobacteria
Spirochaetes
Others
All Bacteria
Alveolates
All Protists
Ascomycetes
All Fungi
Arthropds
Deuterostomes
Nematodes
All Metazoa
Plants
All Eukaryotes
Carbohydrate metabolism
Aminosugars metabolism
Ascorbate and aldarate metabolism
Butanoate metabolism
C5-Branched dibasic acid metabolism
Citrate cycle TCA cycle
Fructose and mannose metabolism
Galactose metabolism
Glycolysis Gluconeogenesis
Glyoxylate and dicarboxylate metabolism
Inositol metabolism
Inositol phosphate metabolism
Nucleotide sugars metabolism
Pentose and glucuronate interconversions
Pentose phosphate pathway
Propanoate metabolism
Pyruvate metabolism
Starch and sucrose metabolism
Energy metabolism
Carbon fixation
Methane metabolism
Nitrogen metabolism
Sulfur metabolism
Reductive carboxylate cycle CO2 fixation
Lipid metabolism
Androgen and estrogen metabolism
Bile acid biosynthesis
C21-Steroid hormone metabolism
Fatty acid biosynthesis .path 1.
Fatty acid biosynthesis .path 2.
Fatty acid metabolism
Glycerolipid metabolism
Phospholipid degradation
Prostaglandin and leukotriene metabolism
Sphingophospholipid biosynthesis
Sterols biosynthesis
Synthesis and degradation of ketone bodies
Nucleotide metabolism
Purine metabolism
Pyrimidine metabolism
Amino acid metabiolism
Alanine and aspartate metabolism
Arginine and proline metabolism
Cysteine metabolism
Glutamate metabolism
Glycine, serine and threonine metabolism
Histidine metabolism
Lysine biosynthesis
Lysine degradation
Methionine metabolism
Phenylalanine metabolism
Phenylalanine, tyrosine and tryptophan biosynthesis
Tryptophan metabolism
Tyrosine metabolism
Urea cycle and metabolism of amino groups
Valine, leucine and isoleucine biosynthesis
Valine, leucine and isoleucine degradation
Other amino acid metaboilism
Aminophosphonate metabolism
beta-Alanine metabolism
Cyanoamino acid metabolism
D-Alanine metabolism
D-Arginine and D-ornithine metabolism
D-Glutamine and D-glutamate metabolism
Glutathione metabolism
Selenoamino acid metabolism
Taurine and hypotaurine metabolism
Glycan metabolism
Blood group glycolipid biosynthesis - lactoseries
Blood group glycolipid biosynthesis - neo-lactoseries
Chondroitin heparan sulfate biosynthesis
Ganglioside biosynthesis
Globoside metabolism
Glycosaminoglycan degradation
Glycosylphosphatidylinositol.GPI.-anchor biosynthesis
Keratan sulfate biosynthesis
Lipopolysaccharide biosynthesis
N-Glycans biosynthesis
O-Glycans biosynthesis
Peptidoglycan biosynthesis
Sphingoglycolipid metabolism
Co-factors and vitamins
Biotin metabolism
Folate biosynthesis
Nicotinate and nicotinamide metabolism
One carbon pool by folate
Pantothenate and CoA biosynthesis
Porphyrin and chlorophyll metabolism
Retinol metabolism
Riboflavin metabolism
Thiamine metabolism
Ubiquinone biosynthesis
Vitamin B6 metabolism
Secondary metabolites
Alkaloid biosynthesis I
Alkaloid biosynthesis II
Clavulanic acid biosynthesis
Diterpenoid biosynthesis
Flavonoids,stilbene and lignin biosynthesis
Indole and ipecac alkaloid biosynthesis
Limonene and pinene degradation
Monoterpenoid biosynthesis
Penicillins and cephalosporins biosynthesis
Polyketide sugar unit biosynthesis
Streptomycin biosynthesis
Terpenoid biosynthesis
Type I polyketide biosynthesis
Xenobiotics
1,1,1-Trichloro-2,2-bis.4-chlorophenyl.ethane_.DDT._degradation
1,2-Dichloroethane degradation
1,4-Dichlorobenzene degradation
2,4-Dichlorobenzoate degradation
3-Chloroacrylic acid degradation
Atrazine degradation
Benzoate degradation via CoA ligation
Benzoate degradation via hydroxylation
Biphenyl degradation
Caprolactam degradation
Carbazole degradation
Ethylbenzene degradation
Fluorene degradation
gamma-Hexachlorocyclohexane degradation
Nitrobenzene degradation
Styrene degradation
Tetrachloroethene degradation
Toluene and xylene degradation

Fig. S4

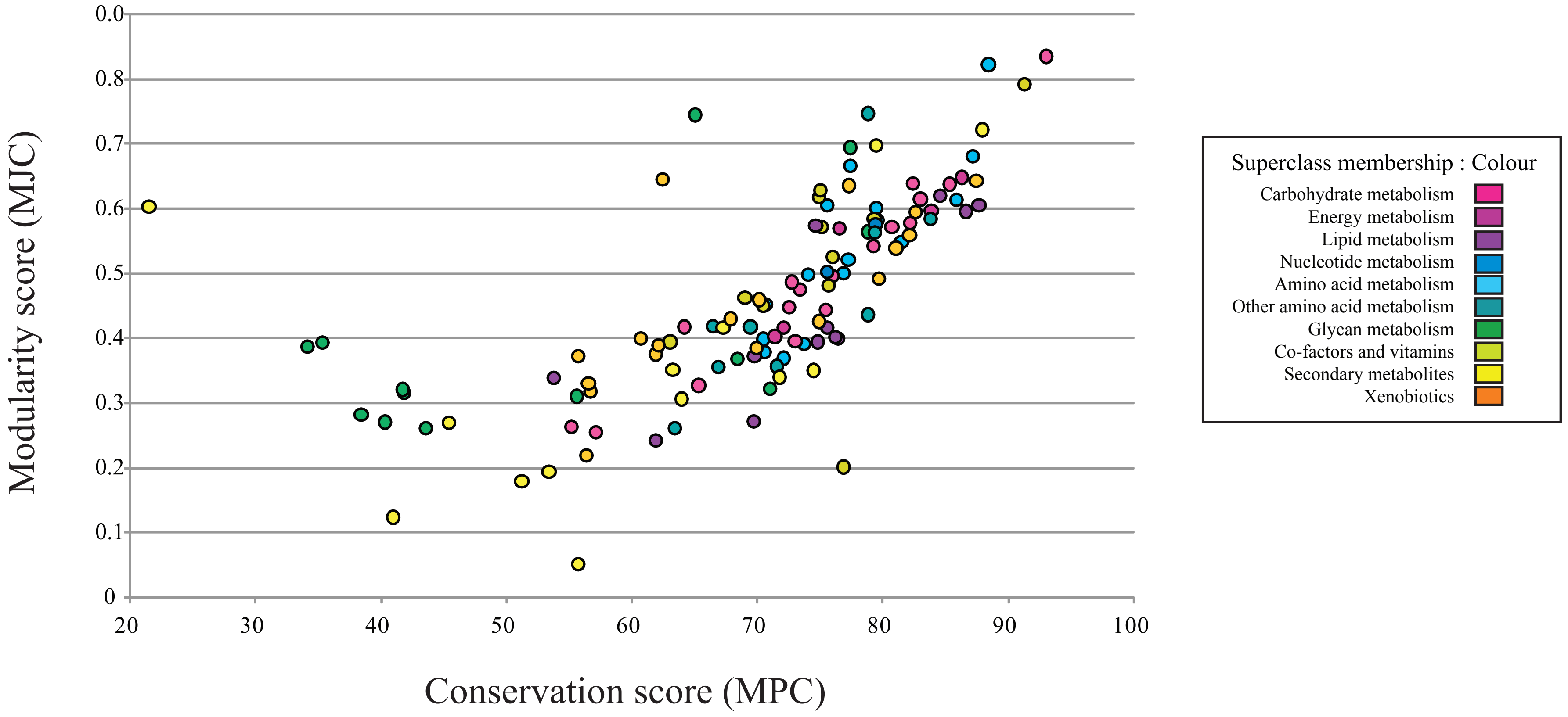

Modularity score (MJC)
0.0
0.8
0.7
0.6
0.5
0.4
0.3
0.2
0.1
0
20
30
40
50
60
70
80
90
100
Conservation score (MPC)
Superclass membership : Colour
Carbohydrate metabolism
Energy metabolism
Lipid metabolism
Nucleotide metabolism
Amino acid metabolism
Other amino acid metabolism
Glycan metabolism
Co-factors and vitamins
Secondary metabolites
Xenobiotics

Fig. S5

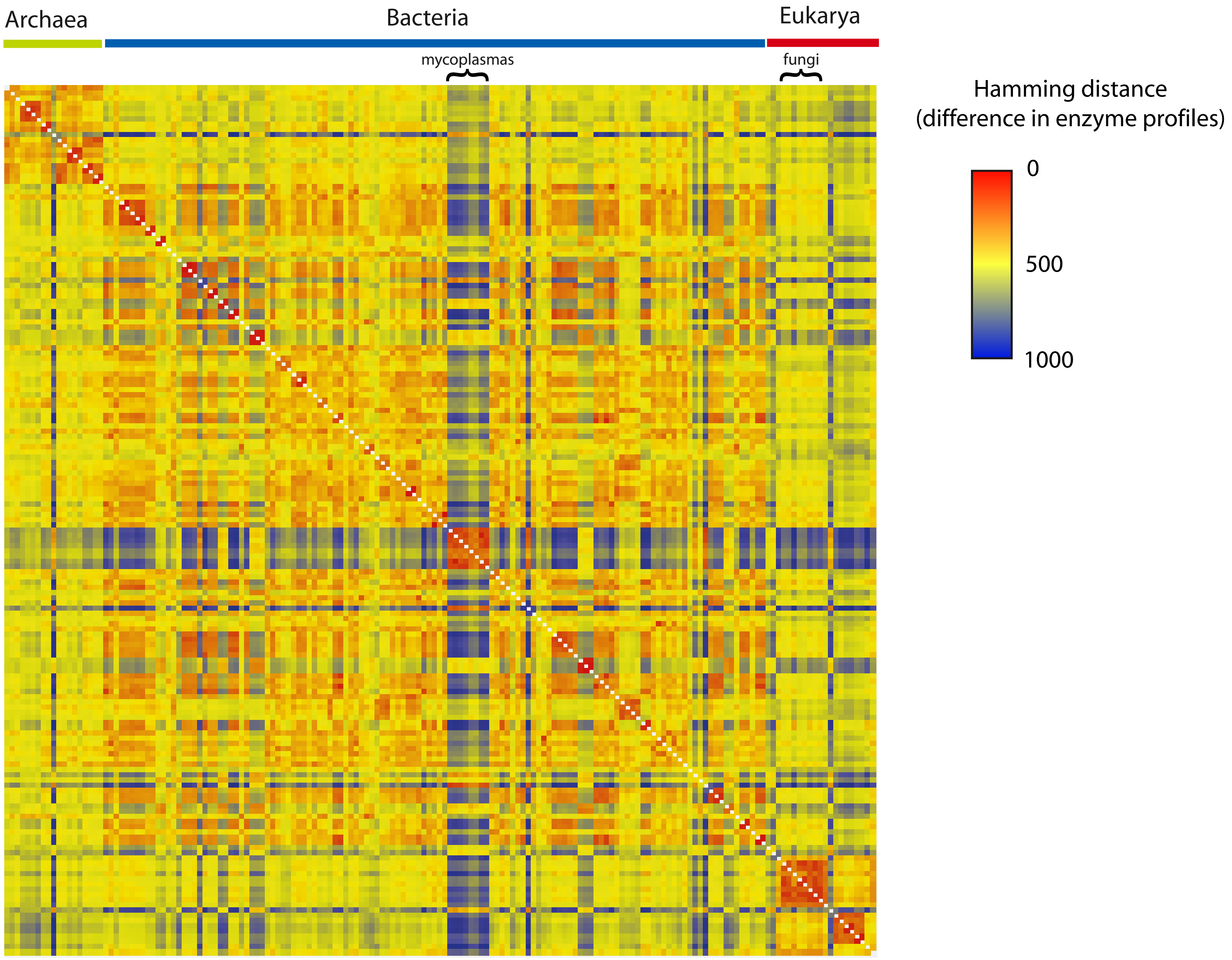
Archaea
Bacteria
Eukarya
mycoplasmas
fungi
Hamming distance
(difference in enzyme profiles)
0
500
1000

Fig. S6

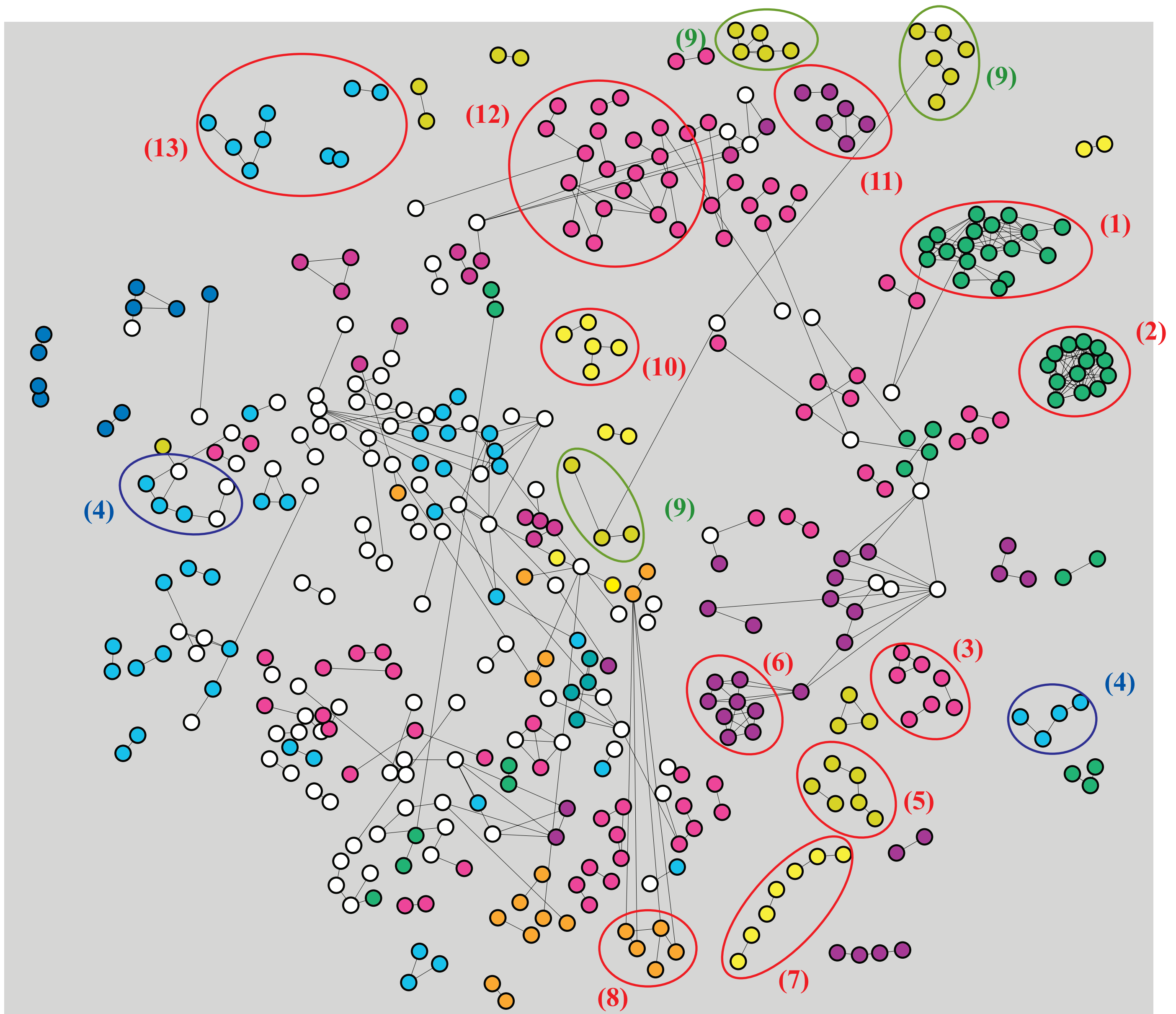

Superclass membership : Node colour	
Carbohydrate metabolism	
Energy metabolism	
Lipid metabolism	
Nucleotide metabolism	
Amino acid metabolism	
Other amino acid metabolism	
Glycan metabolism	
Co-factors and vitamins	
Secondary metabolites	
Xenobiotics	
Multiple superclasses	

Pathway examples

(1) Globoside / Ganglioside metabolism and blood group glycolipid biosynthesis
(2) N-glycan metabolism
(3) Inositol phosphate metabolism
(4) Histidine metabolism
(5) Folate biosynthesis
(6) Prostaglandin and leukotriene metabolism
(7) Diterpenoid biosynthesis
(8) 2,4-Dichlorobenzoate degradation
(9) Porphrin and cholorophyll biosynthesis
(10) Alkaloid biosynthesis I
(11) C21-steroid hormone metabolism
(12) Starch and sucrose metabolism
(13) Phenylalanine, tyrosine and tryptophan biosynthesis

Fig. S7
